# RACK1 enhances STAT3 stability and promotes T follicular helper cell development and function during blood-stage *Plasmodium* infection in mice

**DOI:** 10.1371/journal.ppat.1012352

**Published:** 2024-07-18

**Authors:** Qianqian Cheng, Xiqin Yang, Tao Zou, Lin Sun, Xueting Zhang, Lijiao Deng, Mengyao Wu, Wenbin Gai, Hui Jiang, Tingting Guo, Yuchen Lu, Jie Dong, Chunxiao Niu, Weiqing Pan, Jiyan Zhang

**Affiliations:** 1 Beijing Institute of Basic Medical Sciences, Beijing, China; 2 Shanghai Immune Therapy Institute, Shanghai Jiao Tong University School of Medicine-Affiliated Renji Hospital, Shanghai, China; 3 Department of Tropical Diseases, Navy Medical University, Shanghai, China; 4 Chinese Institute for Brain Research, Beijing, China; University of Melbourne, AUSTRALIA

## Abstract

CD4^+^ T cells are central mediators of protective immunity to blood-stage malaria, particularly for their capacity in orchestrating germinal center reaction and generating parasite-specific high-affinity antibodies. T follicular helper (Tfh) cells are predominant CD4^+^ effector T cell subset implicated in these processes, yet the factors and detailed mechanisms that assist Tfh cell development and function during *Plasmodium* infection are largely undefined. Here we provide evidence that receptor for activated C kinase 1 (RACK1), an adaptor protein of various intracellular signals, is not only important for CD4^+^ T cell expansion as previously implied but also plays a prominent role in Tfh cell differentiation and function during blood-stage *Plasmodium yoelii* 17XNL infection. Consequently, RACK1 in CD4^+^ T cells contributes significantly to germinal center formation, parasite-specific IgG production, and host resistance to the infection. Mechanistic exploration detects specific interaction of RACK1 with STAT3 in *P*. *yoelii* 17XNL-responsive CD4^+^ T cells, ablation of RACK1 leads to defective STAT3 phosphorylation, accompanied by substantially lower amount of STAT3 protein in CD4^+^ T cells, whereas retroviral overexpression of RACK1 or STAT3 in RACK1-deficient CD4^+^ T cells greatly restores STAT3 activity and *Bcl-6* expression under the Tfh polarization condition. Further analyses suggest RACK1 positively regulates STAT3 stability by inhibiting the ubiquitin-proteasomal degradation process, thus promoting optimal STAT3 activity and Bcl-6 induction during Tfh cell differentiation. These findings uncover a novel mechanism by which RACK1 participates in posttranslational regulation of STAT3, Tfh cell differentiation, and subsequent development of anti-*Plasmodium* humoral immunity.

## Author summary

Optimum T cell responses and robust humoral immunity are prerequisites for protection against blood-stage *Plasmodium* infection. CD4^+^ T cells play crucial roles in these processes, not only for the secretion of proinflammatory cytokines but also for their support of antibody production. Defining regulators that manipulate CD4^+^ T cell activities will facilitate the development of optimal interventions against malaria. Previous research has demonstrated the involvement of RACK1 in peripheral T cell homeostasis. However, its *in vivo* role in T cell-mediated immunity to infections remains undefined. In this paper, we investigated the participation of RACK1 in CD4^+^ T cell responses to blood-stage *P*. *yoelii* 17XNL infection. We demonstrate a requirement for RACK1 in the activation and proliferation of *P*. *yoelii*-primed CD4^+^ T cells. More importantly, we elucidate an indispensable role for RACK1 in Tfh cell differentiation and maintenance, which is crucial for germinal center formation, parasite-specific antibody production, and host resistance to *P*. *yoelii* 17XNL infection. Mechanistic studies reveal that RACK1 is essential for maintaining STAT3 stability in *P*. *yoelii*-responsive CD4^+^ T cells, thereby contributing to optimal STAT3 phosphorylation activity and Bcl-6 induction in CD4^+^ T cells during Tfh differentiation. Therefore, we recognize RACK1 as a novel regulator of CD4^+^ T cell effector function and T cell-dependent humoral immunity to blood-stage *Plasmodium* infection.

## Introduction

Malaria, caused by protozoan parasites of the genus *Plasmodium*, remains a devasting infectious disease in tropical and subtropical regions. Despite tremendous progress in reducing the morbidity and mortality, *Plasmodium* infection still causes an estimated 240 million cases and claims more than 600,000 lives annually [1]. Inefficient acquisition of sterile immunity and lack of durable and highly efficacious anti-*Plasmodium* vaccines constitute major obstacles in malaria control [2–4]. Therefore, deeper knowledge of the factors and mechanisms underlying host protective immunity will help improve vaccination and immunotherapeutic strategies to eradicate malaria.

The asexual blood stage of *Plasmodium* infection, which is pathogenic and responsible for all clinical manifestations, has been the focus of investigating naturally acquired or vaccine-elicited anti-malarial immunity. Among the effector mechanisms capable of mediating protection against blood-stage malaria, B cell responses are particularly important in the complete elimination of *Plasmodium* parasites [5–9]. However, parasite-specific high-affinity antibodies are slowly or inadequately generated and easily lost without ongoing infection, and the cellular processes that contribute to the development of humoral immunity remain largely unknown [10–13]. CD4^+^ T cells play an integral role in the acquisition and maintenance of protective immunity to blood-stage malaria [14–17]. Early studies have documented the potential of T helper 1 (Th1) cells in secreting interferon-γ (IFN-γ) and promoting phagocytic cell activities [18–20]. More recently, accumulating evidence suggests that T follicular helper (Tfh) cells induced by *Plasmodium* infection are responsible for B cell secretion of anti-parasitic antibodies in both humans and murine models [21–23]. In addition, there is evidence that severe malaria with compromising IgG responses is largely associated with the disruption of Tfh cell development [24,25]. A clinical trial of the experimental malaria vaccine also confirms the correlation between enhanced Tfh response and improved vaccine efficacy [26]. Accordingly, proper regulation of CD4^+^ T cell activities, especially Tfh cell differentiation and function might facilitate the development of optimal and long-term protective immunity following malaria infection and vaccination.

Tfh is a specialized CD4^+^ T helper (Th) cell subpopulation characterized by expression of the transcription factor B cell lymphoma 6 (Bcl-6), cell surface receptors CXCR5, PD-1, ICOS, signature cytokine IL-21, and serves as key orchestrator of germinal centers (GCs), where they provide critical help to support B cell division, affinity maturation, isotype switching and differentiation into plasma cells (PCs) [27–30]. Generation of the Tfh phenotype is a multifactorial process, requiring extracellular signals from antigens, co-stimulatory molecules, and appropriate cytokine milieu [27,31]. As the lineage-determining transcription factor, Bcl-6 plays a central role in initiating the Tfh program and GC formation. At the same time, Bcl-6 activity is regulated by a complicated network of transcription factors such as B lymphocyte-induced maturation protein 1 (Blimp-1), T cell factor 1 (TCF-1), basic leucine zipper transcription factor (Batf), and those activated by the interplay between specific cytokines and receptors [31–33]. Particularly, IL-6-triggered activation of signal transducers and activators of transcription 3 (STAT3), which is phosphorylated by Janus tyrosine kinases (Jaks) and functions by direct binding to the *Bcl-6* promoter, has been characterized as a crucial determinant of Bcl-6 transcription and Tfh cell differentiation [27,28,34]. The involvement of STAT3 in IL-21 secretion further emphasizes the predominant role of this signaling molecule in Tfh programming and maintenance [35,36]. Moreover, activation of STAT1, which may cooperate with STAT3 to induce Bcl-6 expression, has also been reported to be a potent stimulator of Tfh development during viral infection [37]. On the contrary, IL-2-driven STAT5 activation induces Blimp1, the transcriptional repressor of Bcl-6 function, thereby exerting an inhibitory effect on Tfh generation [28,29,31]. Beyond these findings, additional signaling pathways and specific molecules that may either promote or repress Tfh cell differentiation have been gradually recognized in antigen immunization or viral challenge models [38–39]. However, the molecular requirements and detailed mechanisms underlying the development and regulation of Tfh responses to *Plasmodium* parasites remain to be fully elucidated.

Receptor for activated C kinase 1 (RACK1, encoded by the *Gnb2l1* gene), a well-conserved scaffold protein with seven tryptophan-aspartate 40 (WD40)-repeats, has been originally identified as an adaptor to anchor protein kinase C (PKC) βII [40,41]. By interacting with a variety of signaling molecules, such as transmembrane receptors, tyrosine kinases, and transcription factors, RACK1 serves as an important mediator of intracellular signaling transduction and appears to be a regulator of numerous aspects of cellular activities, including cell proliferation, apoptosis, gene transcription, protein activity, etc. [42–45]. In addition, RACK1 and corresponding pathways also participate in immune responses to extracellular stimuli [46–49]. Our previous study has shown that T cell-targeted deletion of RACK1 led to significantly reduced peripheral CD4^+^ T and CD8^+^ T cells, accompanied by decreased cell proliferation and increased apoptosis upon T cell receptor (TCR) engagement *in vitro* [50]. Therefore, RACK1 may be an important regulator of T cell homeostasis. However, its precise role in T cell effector function *in vivo*, and the impact on T cell-dependent humoral immunity to systemic pathogens have not yet been addressed.

Here we directly addressed the effects of RACK1 on CD4^+^ T cell-mediated immunity to blood-stage *Plasmodium* infection. Using mice that inducibly deleted RACK1 in CD4^+^ T cells and a murine malaria model caused by the self-resolving strain *P*. *yoelii* 17XNL, we found that apart from early activation and expansion of CD4^+^ T cells, RACK1 played an indispensable role in Tfh cell development and function during the infection. Ablation of RACK1 resulted in severely impaired Tfh response, accompanied by defective GC formation and parasite-specific IgG production. Consequently, these RACK1-deficient mice could not recover from the normally non-lethal infection. In mechanism, the lack of RACK1 led to a remarkable decrease in the phosphorylated and total amount of STAT3 protein in CD4^+^ T cells after *P*. *yoelii* infection. This finding was recapitulated in CD4^+^ T cells under the Tfh-like skewing condition, in which the impaired STAT3 activity and Bcl-6 expression could be rectified by enforced expression of RACK1. A similar result was obtained by retroviral overexpression of STAT3 in the Tfh-polarized cells lacking RACK1. Further work demonstrated a crucial role of RACK1 in sustaining STAT3 stability in CD4^+^ T cells, which was probably achieved by inhibiting STAT3 degradation via the ubiquitin-proteasome pathway, Wwp2 and Itch might function as potential ubiquitin ligases in this process. This study provides novel mechanistic insight into posttranslational regulation of STAT3 during Tfh differentiation, the findings expand our understanding of the molecular mechanisms regulating CD4^+^ T cell effector function, as well as the development of protective humoral immunity to blood-stage malaria.

## Results

### Experimental model for exploring effects of RACK1 on CD4^+^ T cell responses to *P*. *yoelii* infection

**Fig 1:**
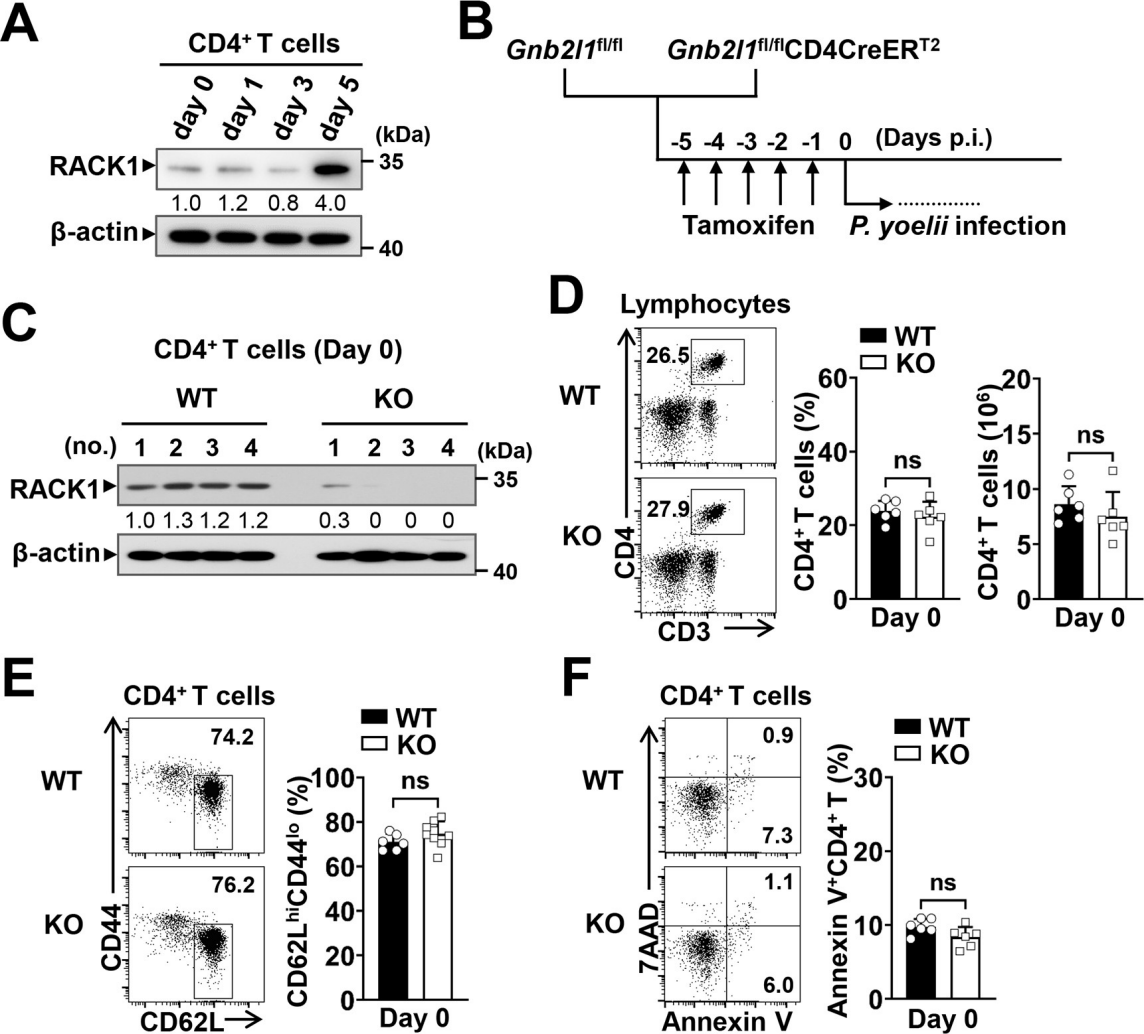
Characteristics of CD4^+^ T cell-targeted RACK1-deficient mice and experimental model of *P*. *yoelii* infection. (A) IB analysis of RACK1 expression in splenic CD4^+^ T cells from wild-type C57BL/6 mice at days 0, 1, 3, 5 of *P*. *yoelii* 17XNL infection. (B) Schematic diagram of the experimental procedure. *Gnb2l1*^fl/fl^CD4CreER^T2^ and *Gnb2l1*^fl/fl^ mice were injected i.p. with 2 mg tamoxifen for consecutive 5 days, 3×10^4^ pRBCs of *P*. *yoelii* 17XNL were inoculated i.p. into these mice on the following day. (C) Validation of RACK1 expression in splenic CD4^+^ T cells from tamoxifen-treated *Gnb2l1*^fl/fl^ (WT) and *Gnb2l1*^fl/fl^CD4CreER^T2^ (KO) mice (n = 4 mice/group). (D) Representative dot plots and bar graphs showing the proportions and absolute numbers of CD4^+^ T (CD3^+^CD4^+^) cells in the spleen of WT and RACK1 KO mice before *P*. *yoelii* 17XNL infection (Day 0). (E) Representative dot plots and bar graph showing the proportions of naïve (CD62L^hi^CD44^lo^) CD4^+^T cells in the spleen of WT and KO mice before *P*. *yoelii* 17XNL infection. (F) Representative dot plots and bar graph showing the frequencies of apoptotic (Annexin V^+^) CD4^+^ T cells in the spleen of WT and KO mice before *P*. *yoelii* 17XNL infection. Data in (A) and (C) are representative of two independent experiments with similar results, numbers indicate quantitative band density normalized to β-actin and are presented relative to that of uninfected mice (day 0) or WT mice. Data in (D-F) are pooled from three independent experiments with 6–9 mice/group and are shown as mean±SD. ns, not significant by Student’s *t* test (D-F).

To explore the functional role of RACK1 in CD4^+^ T cell-mediated immunity to blood-stage malaria, we employed a murine model caused by *P*. *yoelii* 17XNL, in which CD4^+^ T cell-dependent immunity is responsible for full resolution of the infection [22,51]. Firstly, we examined the kinetics of RACK1 expression in CD4^+^ T cells upon parasite challenge. C57BL/6 mice were inoculated intraperitoneally (i.p.) with 3×10^4^ parasitized erythrocytes (pRBCs). As shown in Fig 1A, the protein level of RACK1 was strikingly elevated in splenic CD4^+^ T cells within 5 days of infection, implying a biological relevance of this molecule with CD4^+^ T cell activities and functions. Our previous work suggested that loss of RACK1 in T cells resulted in a decrease in peripheral CD4^+^ and CD8^+^ T cells [50]. Herein, to exclude the initial defects in peripheral T cell homeostasis, we crossed *Gnb2l1*^fl/fl^ mice with the CD4CreER^T2^ transgenic strain to generate *Gnb2l1*^fl/fl^CD4CreER^T2^ mice, which allows for inducible deletion of *loxP*-flanked *Gnb2l1* alleles (encoding RACK1) in peripheral CD4^+^ T cells by tamoxifen administration [52]. As confirmed by IB analysis, deficiency of RACK1 in splenic CD4^+^ T cells was achieved by i.p. injection with tamoxifen for consecutive 5 days. For blood-stage *Plasmodium* infection, tamoxifen-treated Gnb*2l1*^fl/fl^CD4CreER^T2^ (designed as RACK1 KO mice) and *Gnb2l1*^fl/fl^ littermates (designed as WT mice) were infected with 3×10^4^ pRBCs of *P*. *yoelii* 17XNL on the following day (Fig 1B and 1C). We then tested whether inducible deletion of RACK1 influenced peripheral CD4^+^ T cells under steady-state conditions. As anticipated, the percentage and absolute number of splenic CD4^+^ T cells in RACK1 KO mice were comparable to those in WT littermate controls (Fig 1D). A similar phenomenon was also found in other T cell subsets including CD8^+^ T, NKT, and γδ T populations in the spleen (S1A–S1C Fig). Moreover, tamoxifen-mediated RACK1 deficiency did not alter basal activation and apoptosis of CD4^+^ T cells (Fig 1E and 1F), as well as the initial activation of CD8^+^ T cells in the spleen (S1D Fig). Therefore, this CD4^+^ T cell-targeted RACK1-deficient model may provide an excellent tool to investigate the *in vivo* role of RACK1 and corresponding signals in CD4^+^ T cell responses to *Plasmodium* infection.

### RACK1 promotes CD4^+^ T cell activation and expansion upon blood-stage *P*. *yoelii* 17XNL infection

**Fig 2:**
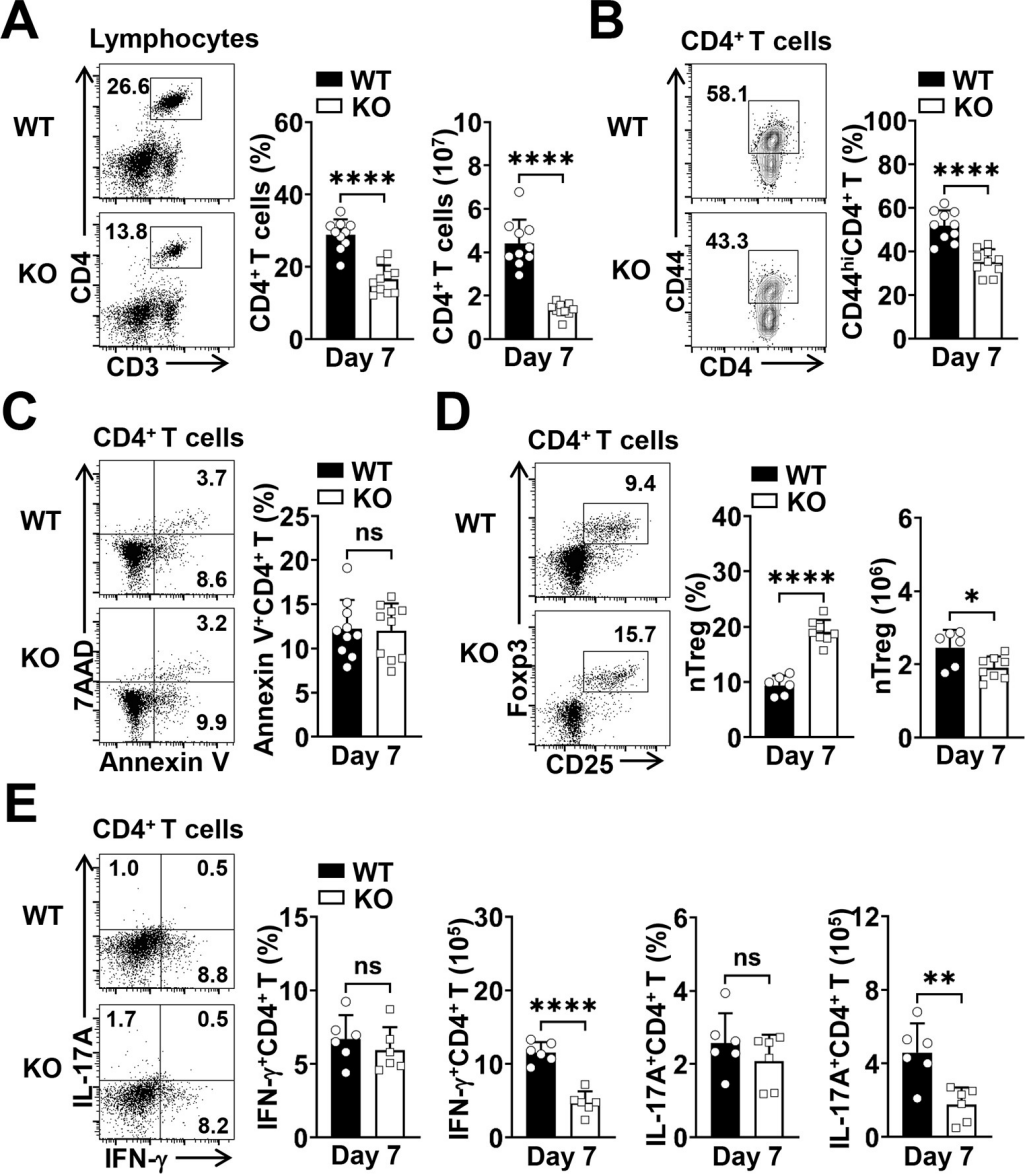
RACK1 promotes activation and expansion of CD4^+^ T cells upon *P*. *yoelii* 17XNL infection. Tamoxifen-treated WT and RACK1 KO mice were challenged i.p. with 3×10^4^ pRBCs of *P*. *yoelii* 17XNL. (A) Representative dot plots and bar graphs showing the proportions and numbers of CD4^+^ T cells in the spleen of WT and KO mice at day 7 p.i. (B) Representative counter plots and bar graph showing the proportions of activated (CD44^hi^) CD4^+^ T cells in the spleen of WT and KO mice at day 7 p.i. (C) Representative dot plots and bar graph showing the frequencies of apoptotic (Annexin V^+^) CD4^+^ T cells in the spleen of WT and KO mice at day 7 p.i. (D) Representative dot plots and bar graphs showing the proportions (among CD4^+^ T cells) and numbers of nTreg (CD25^+^Foxp3^+^CD4^+^) cells in the spleen of WT and KO mice at day 7 p.i. (E) Splenocytes from WT and RACK1 KO mice at day 7 p.i. were stimulated *ex vivo* with PMA/ionomycin in the presence of brefeldin A for 5 h. Representative dot plots and bar graphs showing IFN-γ and IL-17A production by CD4^+^ T cells. Data are pooled from two or four experiments with 6–10 mice/group and are shown as mean±SD. *P<0.05, **P<0.01, ****P<0.0001, and ns, not significant by Student’s *t* test (A-E).

Next, we dissected the effects of RACK1 on CD4^+^ T cell responses after *P*. *yoelii* 17XNL infection. As described above, tamoxifen-treated RACK1 KO mice and WT littermates were infected with 3×10^4^ pRBCs of *P*. *yoelii* 17XNL. Consistent with the requirement for RACK1 in conventional T cell proliferation *in vitro* [50], we observed that deletion of RACK1 led to a remarkably reduced proportion and absolute number of splenic CD4^+^ T cells at day 7 post-infection (p.i.) (Fig 2A). In parallel with this, the percentage of activated CD44^hi^CD4^+^ T cells was lower in the spleen compared with that in WT mice (Fig 2B), while apoptotic CD4^+^ T cells did not appear to be significantly different between the two groups of mice at day 7 p.i., as assessed by AnnexinV/7AAD dual staining (Fig 2C). Moreover, although contracted CD4^+^ T cell population was found as the infection progressed (e.g., day 16 p.i.), RACK1 KO mice exhibited a persistently lower proportion and overall number of CD4^+^ T cells in the spleen, as compared with those in WT mice (S2A Fig). By contrast, in line with the notion that RACK1 depletion was achieved specifically in CD4^+^ T cells, the expansion of CD8^+^ T and innate-like T cell populations was not significantly affected in response to parasite challenge (S2B Fig).

Given the implication of effector and regulatory T cells in the early control of *Plasmodium* infection [53,54], we further tested whether loss of RACK1 influenced the emergence of these cell populations in response to *P*. *yoelii* 17XNL. As shown in Fig 2D, a higher percentage of natural regulatory T (nTreg, CD4^+^CD25^+^Foxp3^+^) cells was noted in RACK1 KO mice at day 7 p.i., however, due to the reduction of total splenic CD4^+^ T cells, the cell number was lower than that in WT mice. Meanwhile, to assess the development of classical effector CD4^+^ T cells (e.g., Th1, Th17), splenocytes were stimulated *ex vivo* and examined for their potential to secrete the lineage-associated cytokines. As expected, the numbers of IFN-γ- or IL-17A-producing CD4^+^ T cells were significantly reduced by the absence of RACK1, even though equivalent proportions of these cell subsets were found in WT and RACK1 KO mice at day 7 p.i. (Fig 2E).

Together, these data demonstrate that RACK1 is required for *P*. *yoelii*-primed activation and expansion of CD4^+^ T cells, and exerts a beneficial effect on Th1, Th17, and nTreg cell expansion during the early phase of infection.

### RACK1 is indispensable for optimal Tfh cell response during blood-stage *P*. *yoelii* 17XNL infection

**Fig 3:**
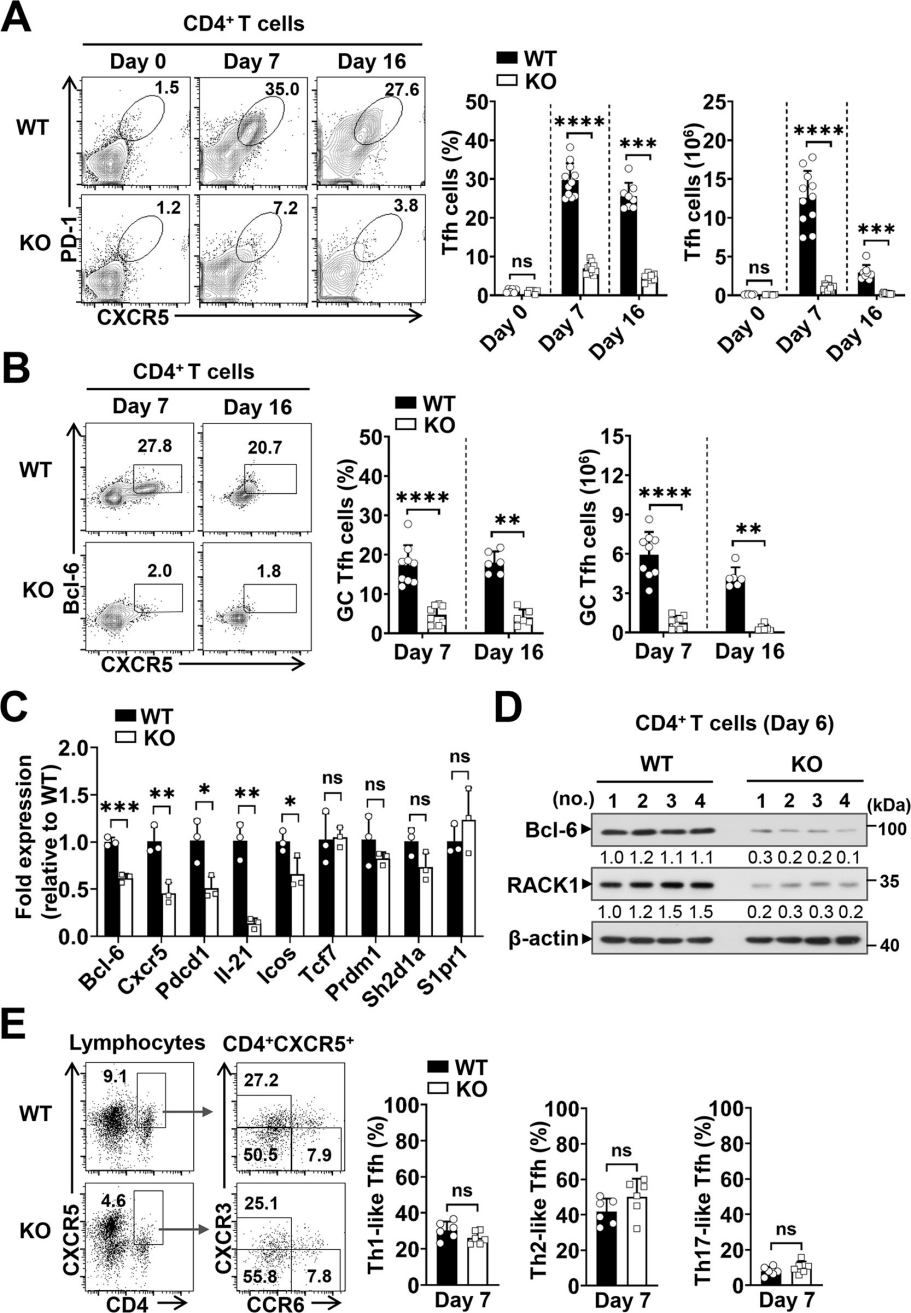
RACK1 is indispensable for Tfh cell development during blood-stage *P*. *yoelii* 17XNL infection. (A) Representative counter plots and bar graphs showing the proportions and numbers of Tfh (CD4^+^CXCR5^+^PD-1^+^) cells in the spleen of WT and RACK1 KO mice at day 0, day 7, and day 16 p.i. (B) Representative counter plots and bar graphs showing the proportions and numbers of GC Tfh (CD4^+^CXCR5^+^Bcl-6^hi^) cells in the spleen of WT and KO mice at day 7 and day 16 p.i. (C) Quantitative RT-PCR analysis of Tfh-lineage signature genes *Bcl-6*, *Cxcr5*, *Pdcd1*, *Il-21*, *Icos* and special regulatory genes *Tcf7*, *Prdm1*, *sh2d1a*, *S1pr1* in splenic CD4^+^ T cells from WT and KO mice at day 6 p.i., bar graph showing gene expression level normalized to *β-actin*, relative to that of WT mice. (D) IB analysis of Bcl-6 expression in splenic CD4^+^ T cells from WT and KO mice at day 6 p.i. (n = 4 mice/group). Numbers indicate densitometry of the bands normalized to *β-actin*, relative to that of WT mice. (E) Gating strategy of CXCR3, CCR6 expression on CXCR5^+^CD4^+^ T cells, bar graph showing the proportions of Th1-like (CXCR3^+^CCR6^-^), Th2-like (CXCR3^-^CCR6^-^) and Th17-like (CXCR3^-^CCR6^+^) Tfh subsets in the spleen of WT and KO mice at day 7 p.i. Data are from three or four experiments with 6–12 mice/group (A, B) or representative of two replicate experiments with 3–6 mice/group (C-E). Data are shown as mean±SD. *P<0.05, **P<0.01, ***P<0.001, ****P<0.0001 and ns, not significant by Mann-Whitney test (A, B) or Student’s *t* test (C, E).

Recent work has highlighted the importance of Tfh cells in immune resistance to blood-stage malaria [21–25], we then asked whether lack of RACK1 in CD4^+^ T cells resulted in phenotypic abnormality of the Tfh compartment. To this end, Tfh cell development following *P*. *yoelii* 17XNL infection was analyzed by flow cytometry. Despite comparable basal levels in steady state, WT mice exhibited greatly increased Tfh cells (CXCR5^+^PD-1^+^) in the spleen at day 7 p.i., whereas RACK1 KO mice developed a proportional decrease of the Tfh population among total CD4^+^ T cells (7.0±1.3% versus 29.7±4.2%), in combination with decreased CD4^+^ T cells in these mice, the absolute number of Tfh cells was almost reduced to ~8.8% of that in WT littermate controls (Fig 3A). Furthermore, although a slight decrease of Tfh cells was present in both groups of mice at day 16 p.i., the proportion and cell number were still substantially lower in RACK1 KO mice than those seen in WT mice (Fig 3A). To further confirm the effects of RACK1 on the Tfh phenotype, we examined the induction of fully polarized germinal center (GC) Tfh cells. As anticipated, the percentage and absolute number of CXCR5^+^Bcl-6^hi^ GC Tfh cells were remarkably decreased in RACK1 KO mice compared to those in WT mice at day 7 and day 16 p.i. (Fig 3B). Therefore, RACK1 may participate in Tfh cell development and function during *P*. *yoelii* infection. In line with these observations, quantitative RT-PCR detected substantially lower levels of Tfh signature molecules, including Bcl-6, CXCR5, PD-1 (encoded by *Pdcd1*), ICOS, and the hallmark cytokine Il-21 mRNAs in splenic CD4^+^ T cells from RACK1 KO mice at day 6 p.i., whereas the expression of regulators that known to promote or repress the Tfh program [31,33], such as TCF-1 (encoded by *Tcf7*), Blimp-1 (encoded by *Prdm1*), SAP (encode by *sh2d1a*) and S1PR1 (encoded by *S1pr1*), was not significantly different in RACK1-deficient cells and the WT controls (Fig 3C). Consistently, RACK1 KO mice exhibited a much lower amount of Bcl-6 protein in splenic CD4^+^ T cells, as compared with that in WT mice at day 6 p.i. (Fig 3D). Moreover, the similar frequencies of Annexin V^+^ Tfh cells in the spleen of WT and RACK1 KO mice supported the view that RACK1 promotes initial differentiation of Tfh cells without affecting cell viability in response to *P*. *yoelii* 17XNL (S3A Fig).

Emerging evidence has reported that Tfh cells comprise functionally distinct populations (i.e., Th1-, Th2-, Th17-like) based on their differential phenotypes and capacity to help B cells [55,56], we next evaluated the development of these Tfh populations by assessing cell surface CXCR3 and CCR6 expression. As shown in Fig 3E, no significant differences in the proportions of Th1-like (CXCR3^+^CCR6^-^), Th2-like (CXCR3^-^CCR6^-^), or Th17-like (CXCR3^-^CCR6^+^) Tfh subsets among total CD4^+^CXCR5^+^ cells were found in WT and RACK1 KO mice at day 7 p.i., likewise, a similar trend in the development of these Tfh cell populations were observed at day 16 p.i. (S3B Fig), indicating that RACK1 exerts beneficial effect on global Tfh cell development, rather than driving either helper lineage skewed population.

Collectively, these data highlight an indispensable role for RACK1 in Tfh cell differentiation and maintenance, which may favor the development of humoral immune responses to blood-stage *Plasmodium* infection.

### RACK1 in CD4^+^ T cells is essential for GC-dependent humoral immunity and resolution of *P*. *yoelii* infection

**Fig 4:**
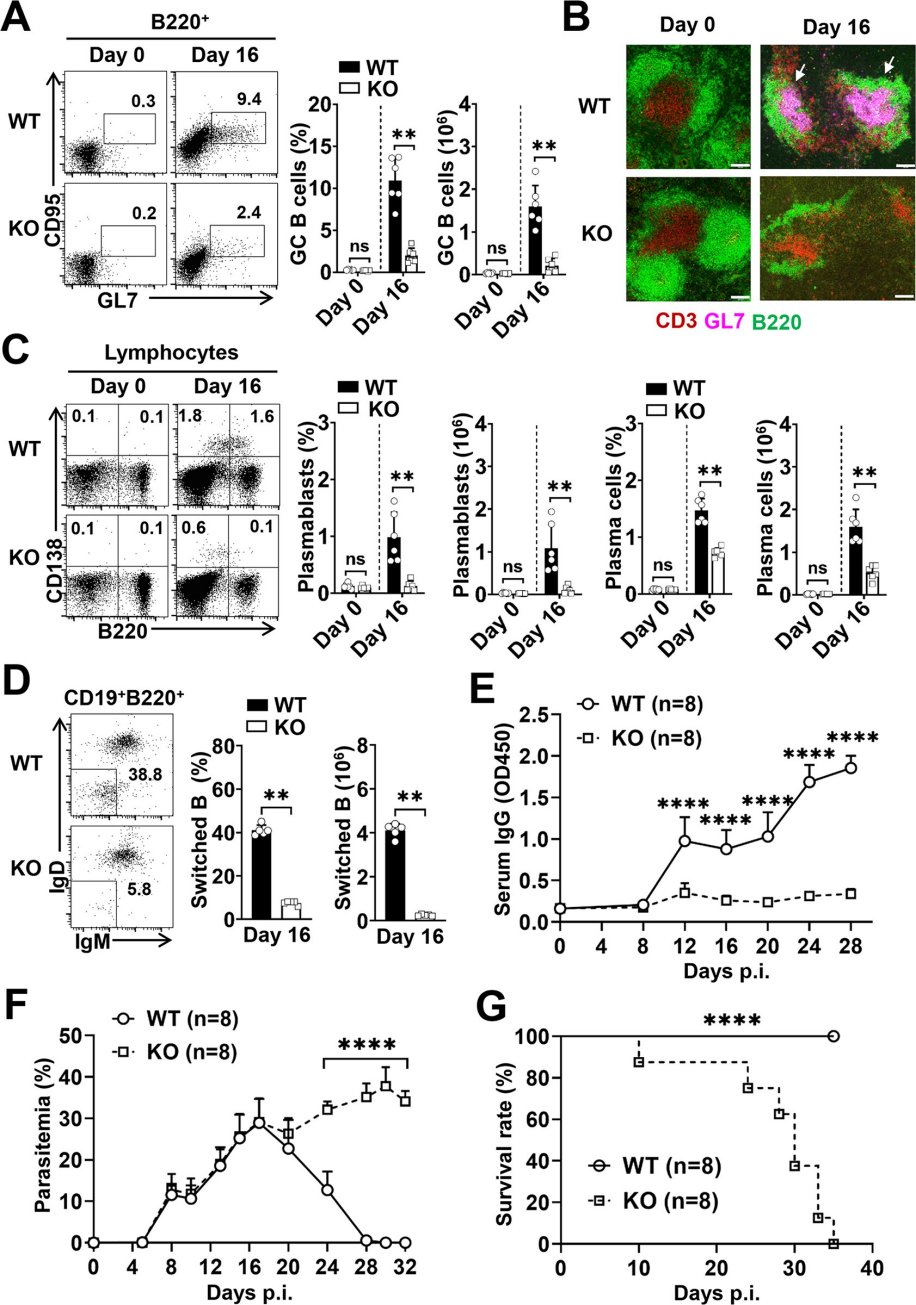
RACK1 in CD4^+^ T cells is essential for GC formation and resolution of the infection. (A) Representative dot plots and bar graphs showing the proportions and numbers of GC B (GL-7^+^CD95^+^B220^+^) cells in the spleen of WT and RACK1 KO mice at day 0 and day 16 p.i. (B) Representative spleen sections of day 0 and day 16-infected WT and KO mice, identifying B cell follicles with anti-B220 (green), T cell zones with anti-CD3 (red), and germinal centers with anti-GL7 (pink) staining. Arrows denote individual examples of GL7^+^ GC structures. Scale bars, 100 μm. (C) Representative dot plots and bar graphs showing the proportions and numbers of plasmablasts (CD138^+^B220^hi^) and plasma (CD138^+^B220^lo^) cells in the spleen of WT and KO mice at day 0 and day 16 p.i. (D) Representative dot plots and bar graphs showing the proportions and numbers of Ig-switched GC B (IgD^lo^IgM^lo^B220^+^CD19^+^) cells in the spleen of WT and KO mice at day 16 p.i. (E) Serum levels of parasite-specific IgG in WT and KO mice throughout *P*. *yoelii* 17XNL infection. Data are expressed as relative OD450 values. (F) Parasitemia (% infected erythrocytes) kinetics and (G) overall survival of WT and KO mice infected with *P*. *yoelii* 17XNL. Data are pooled from two or three experiments with 5–6 mice/group (A-D) or representative of two replicate experiments with similar results (E-G, n = 8 mice/group). Data are shown as mean±SD. **P<0.01, ****P<0.0001 by Mann-Whitney test (A, C, D), Student’s *t* test (B), two-way ANOVA (E, F), or log-rank test (G).

The functional properties of Tfh cells in orchestrating germinal center (GC)-dependent humoral immunity prompted us to investigate whether loss of RACK1 constrains *P*. *yoelii* 17XNL-derived GC reaction. As expected, in contrast to WT mice, which developed a pronounced GC B cell population (B220^+^GL7^+^CD95^+^) at day 16 p.i., over 80% decrease in proportion and a ~6.5-fold reduction in absolute number of GC B cells were found in the spleen of RACK1 KO mice, as assessed by flow cytometry (Fig 4A). Furthermore, the histological examination demonstrated that, although normal structures of B cell follicles (B220^+^) and T cell zones (CD3^+^) were observed in RACK1 KO mice prior to *P*. *yoelii* infection (Figs 4B and S4A), in comparison with the abundance of typical GCs (GL7^+^) that developed in WT mice at day 16 p.i., RACK1 KO mice displayed profoundly impaired architecture of B cell follicles with extremely fewer and smaller GC areas, along with significantly reduced T cell zones in the spleen (Figs 4B and S4B–S4F). In concert with these findings, RACK1 KO mice also exhibited substantially decreased proportions and total numbers of plasmablasts (CD138^+^B220^hi^), plasma cells (CD138^+^B220^lo^), as well as Ig-class switched GC B cells (B220^+^CD19^+^IgM^lo^IgD^lo^) at this time (Fig 4C and 4D). Correspondingly, in comparison with the dramatically increased parasite-specific IgG in WT mice from day 16 p.i. onward, RACK1 KO mice developed slightly elevated IgG in the serum after *P*. *yoelii* challenge, the levels were extremely lower than those detected in WT mice (Fig 4E). As such, targeted deletion of RACK1 in CD4^+^ T cells severely perturbs GC formation and anti-*Plasmodium* humoral immunity.

Given that the newly described subpopulation of follicular T cells, named T follicular regulatory (Tfr) cells, play an essential role in suppressing Tfh-dependent GC formation and protective immunity to blood-stage malaria [57,58], we then assessed whether the impaired Tfh-dependent GC reaction in RACK1 KO mice was associated with Tfr response elicited by *P*. *yoelii* 17XNL. As shown in S3C Fig, total splenic Tfr cells, defined as the Foxp3^+^CXCR5^+^CD4^+^ T population, were equivalent between the two groups at day 7 p.i., even though a higher percentage was found in RACK1 KO mice primarily due to remarkably reduced CD4^+^ T cells compared to WT littermates, suggesting that the defective Tfh functionality is not attributed to inhibition by Foxp3^+^ Tfr cells.

As a consequence, when evaluating the biological relevance of CD4^+^ T cell-targeted RACK1 deficiency in host resistance to *P*. *yoelii* 17XNL infection, we noted that coinciding with the steadily ascending parasite-specific IgG, WT mice developed a peak parasitemia (~28.95%) at day 17 p.i., the parasite burdens were gradually declined afterward, and 100% of mice were fully recovered from the infection within 30 days. By contrast, RACK1 KO mice displayed a similar peak parasitemia at day 17 p.i., but suffered from unremitting hyperparasitemia at the later stage, and all succumbed to the infection by day 35 p.i. (Fig 4F and 4G). Accordingly, RACK1 in CD4^+^ T cells is critical for generating adequate B cell responses to eradicate blood-stage *P*. *yoelii* infection.

### RACK1 positively regulates the protein level and phosphorylation of STAT3 in CD4^+^ T cells during *P*. *yoelii* infection

**Fig 5:**
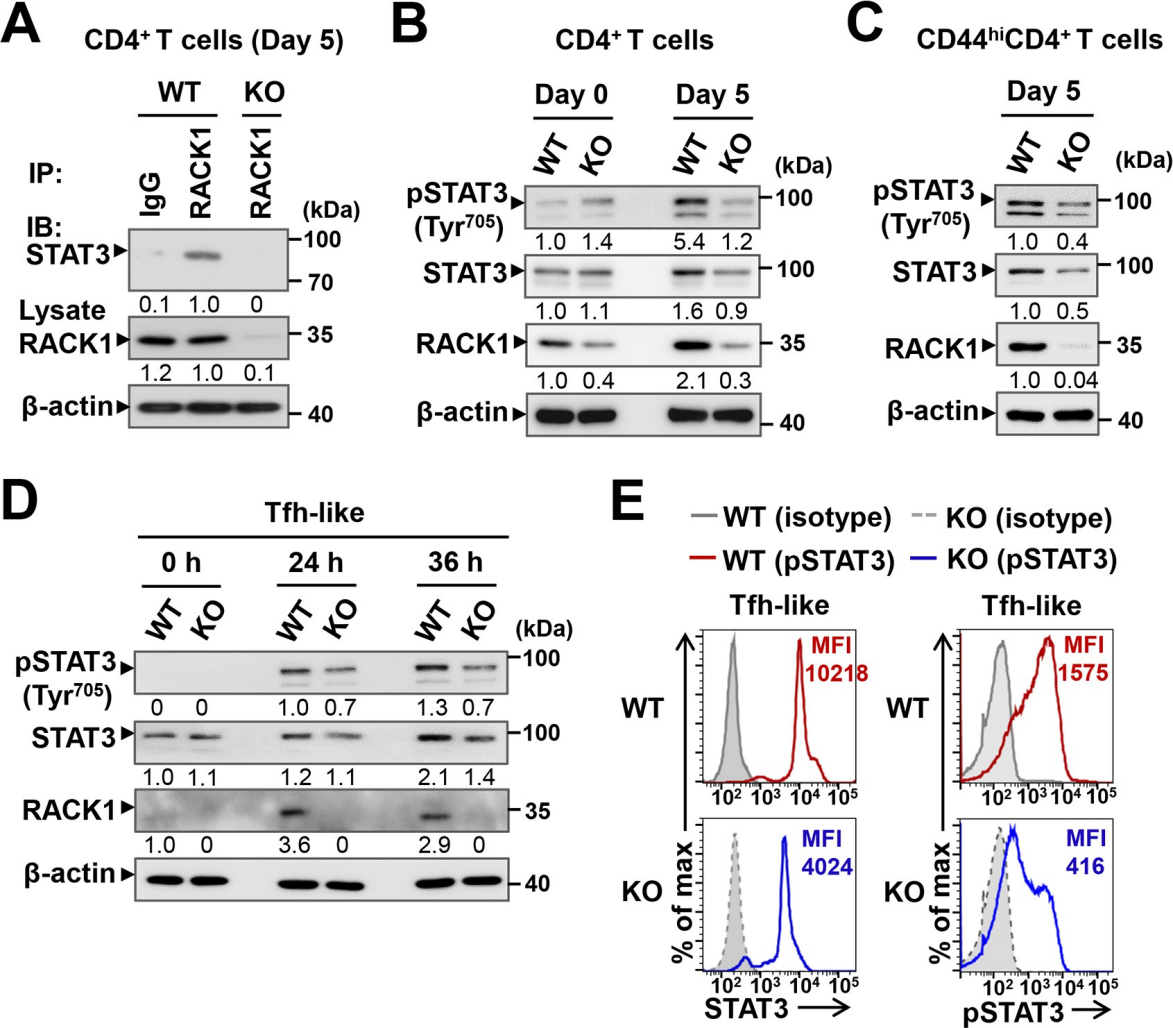
RACK1 positively regulates the protein level and phosphorylation activity of STAT3 in CD4^+^ T cells. (A) Co-IP analysis of endogenous interaction between RACK1 and STAT3. CD4^+^ T cell lysates from *P*. *yoelii* 17XNL-infected WT and RACK1 KO mice at day 5 p.i. were prepared for IP with anti-RACK1 (or IgG control) antibody, followed by IB with anti-STAT3 antibody. Lysates without IP were subjected to IB for RACK1 expression. (B) IB analysis of pSTAT3 (Tyr^705^) and STAT3 protein in splenic CD4^+^ T cells from WT and KO mice at day 0 and day 5 p.i., numbers indicate densitometry of the bands relative to that of uninfected WT mice. (C) IB analysis of pSTAT3 (Tyr^705^) and STAT3 protein in activated CD44^hi^CD62L^lo^CD4^+^ T cells from WT and RACK1 KO mice at day 5 p.i. (D) Naïve CD4^+^T cells sorted from the spleen of WT and RACK1 KO mice were stimulated with PMA/ionomycin and cultured under the Tfh-like polarizing condition for 0, 24, 36 h, pSTAT3 (Tyr^705^) and STAT3 protein in the Tfh-differentiated cells were assessed by IB analysis. (E) Naïve CD4^+^ T cells from WT (red line) and RACK1 KO (blue line) mice were cultured under the Tfh-like skewing condition for 36 h, representative histograms showing the median fluorescent intensity (MFI) of STAT3 protein and pSTAT3 (Tyr^705^) in Tfh-like differentiated CD4^+^ T cells, obtained by intracellular staining and phosflow analysis, respectively. The gray plots depict isotype IgG staining controls. Data are representative of two or three independent experiments with similar results.

Numerous studies have elucidated the scaffolding properties of RACK1 and its functional role in intracellular signaling pathways [42–45]. Herein, to delineate the underlying mechanism by which RACK1 regulates Tfh cell development and function, we aimed to find possible signaling molecules that interact with RACK1. To this end, splenic CD4^+^T cells from naïve and *P*. *yoelii*-infected mice were prepared for immunoprecipitation with an anti-RACK1 antibody and then subjected to mass spectrometric analysis. The result identified that STAT3, a well-established regulator upstream of Bcl-6 [34,35], was present in the RACK1-immunoprecipitated samples obtained from day 5-infected WT mice (S5 Fig). Co-immunoprecipitation (Co-IP) analysis further confirmed endogenous conjugation of RACK1 with STAT3 in CD4^+^ T cells from WT mice, which was absent in the RACK1-deficient cells at day 5 p.i. (Fig 5A). Based on this finding, we then examined the phosphorylation activity of STAT3 and other signaling pathways that might be implicated in T cell responses to extracellular stimuli [59,60]. As anticipated, a clear reduction in tyrosine-phosphorylated STAT3 (pSTAT3, Tyr^705^) was shown in CD4^+^ T cells from day 5-infected RACK1 KO mice (Fig 5B). In parallel with this, a greatly decreased accumulation of STAT3 protein was found in RACK1-deficient CD4^+^ T cells compared to their WT counterparts (Fig 5B). Previous work has suggested a cooperative role for STAT1 and the antagonistic role for STAT5 in STAT3-mediated regulation of Bcl-6 induction and Tfh priming [29,31,37], we therefore asked whether these transcription factors were similarly affected by the absence of RACK1. In contrast to the markedly reduced protein level and phosphorylation of STAT3, the protein amounts of STAT1 and STAT5 were relatively normal in RACK1-deficient CD4^+^ T cells at day 5 p.i., although a slightly decreased pSTAT1 (Tyr^701^) was noted in comparison with the WT controls (S6A and S6B Fig). In addition, no difference in the activation of ERK1/2 and p38 MAPK pathways downstream of TCR signaling was observed in CD4^+^ T cells from two groups of mice early after the infection (S6C Fig). These data support a possible link between RACK1 and STAT3 in *P*. *yoelii*-primed Tfh cell differentiation.

Furthermore, considering that RACK1 deficiency resulted in defective CD4^+^ T cell activation upon the parasite challenge (Fig 2B), we wondered whether the impaired STAT3 activity was due to a CD4^+^ T cell activation defect in RACK1 KO mice. To exclude this possibility, we next examined protein expression and phosphorylation of STAT3 in activated CD44^hi^CD62L^lo^CD4^+^T cells. Consistent with the observations in CD4^+^ T cells at day 5 p.i., the phosphorylated and total amount of STAT3 were reduced to the same extent in activated CD4^+^ T cells deficient for RACK1, as compared with those in their WT counterparts (Fig 5C). Therefore, RACK1 might contribute to sustaining optimal protein level and phosphorylation activity of STAT3 in CD4^+^ T cells following *P*. *yoelii* infection.

Additionally, the positive role of RACK1 in regulating STAT3 activity was further confirmed when we monitored the capacity of CD4^+^ T cells to differentiate into Tfh-like cells *in vitro*. For this purpose, naïve CD4^+^ T cells sorted from WT and RACK1 KO mice were stimulated and cultured under the condition known to efficiently induce Tfh-like cells, which closely mimics the transcriptional programs of *in vivo*-generated Tfh cells [39]. Consistent with *P*. *yoelii* infection, significantly lower protein amount and phosphorylation of STAT3 in the Tfh-polarized CD4^+^ T cells lacking RACK1 were detected by IB analysis within 36 h of culture (Fig 5D). Concurrently, a remarkable decrease in the median fluorescent intensity (MFI) of STAT3 protein and pSTAT3 (Tyr^705^) was observed in the RACK1-deficient cells compared to their WT counterparts, as assessed by intracellular staining and phosflow analysis (Fig 5E). Therefore, RACK1 appears to play a similar role in *P*. *yoelii*-primed Tfh differentiation and the *in vitro* polarization program, which strongly suggests that RACK1 is required to allow optimal protein amount and phosphorylation activity of STAT3 in CD4^+^ T cells during Tfh cell development.

### Effect of RACK1 on STAT3 activity is responsible for Bcl-6 expression during Tfh cell differentiation

**Fig 6:**
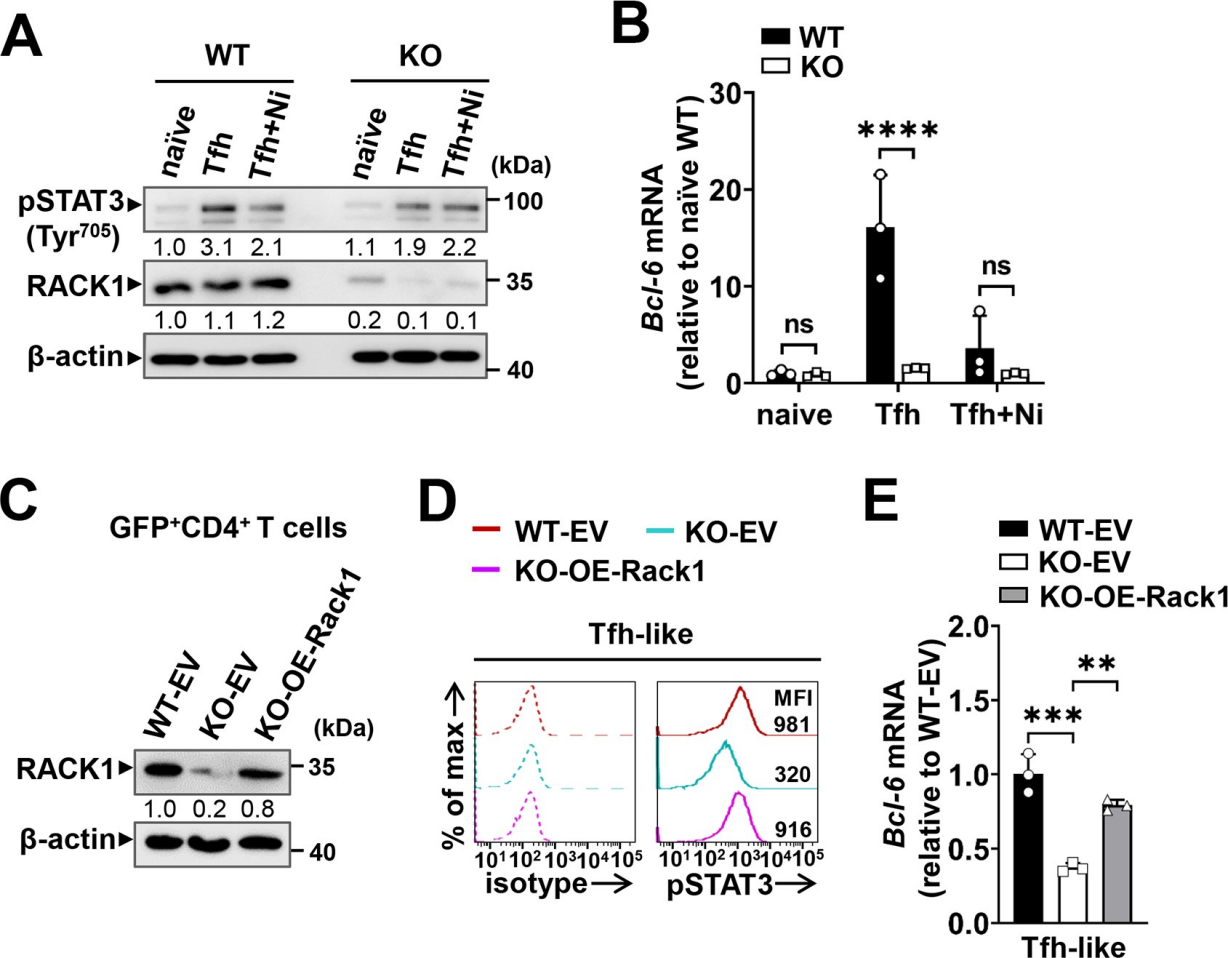
Effect of RACK1 on STAT3 activity is critical for Bcl-6 expression during Tfh differentiation. (A) Naïve CD4^+^ T cells from WT and RACK1 KO mice were cultured under the Tfh-like skewing condition for 3 days, with or without adding 1μM STAT3 activity inhibitor, niclosamide (Ni). The pSTAT3 (Tyr^705^) levels in naïve and Tfh-like differentiated cells were examined by IB analysis, numbers indicate densitometry of the bands relative to that of naïve WT cells. (B) Quantitative RT-PCR analysis of Bcl-6 expression in naïve and Tfh-like differentiated CD4^+^ T cells as described in (A) (n = 3 mice/group). (C-E) Naïve CD4^+^ T cells sorted from WT and RACK1 KO mice were pre-activated and spin infected with the retrovirus expressing RACK1 (OE-Rack1) or GFP alone (EV), the transduced (GFP^+^) cells were then sorted and polarized under the Tfh-like condition for 3 days. (C) Validation of RACK1 expression in retrovirally transduced GFP^+^CD4^+^ T cells, numbers indicate densitometry of the bands relative to that of WT cells transduced with EV. (D) Phosflow analysis of pSTAT3 (Tyr^705^) MFI in Tfh-like differentiated cells (36 h) transduced with the OE-Rack1 or EV vectors, the dash lines depict isotype IgG staining controls. (E) Quantitative RT-PCR for Bcl-6 expression in Tfh-like differentiated cells (3 days) transduced with the OE-Rack1 or EV vectors, data shown are Bcl-6 mRNA levels normalized to *β-actin*, relative to that of WT cells transduced with EV. Data are representative of two independent experiments and are shown as mean±SD. **P<0.01, ***P<0.001, ****P<0.0001 and ns, not significant by two-way ANOVA with Bonferroni’s test (B) or one-way ANOVA with Tukey’s test (E).

As tyrosine phosphorylation of STAT3 is important for Bcl-6 induction and Tfh differentiation [28,34], we next utilized the Tfh polarization system to address whether RACK1 promotes Bcl-6 expression via regulating STAT3 activity. For this, naïve CD4^+^ T cells from WT and RACK1 KO mice were cultured under the Tfh-like skewing condition for 3 days in the presence or absence of niclosamide, a specific inhibitor of STAT3 activity [61]. As confirmed by IB analysis, pSTAT3 (Tyr^705^) was upregulated in the Tfh-polarized cells, and the level in RACK1-deficient cells was much lower than that in their WT counterparts (Fig 6A). Meanwhile, quantitative RT-PCR analysis detected extremely lower Bcl-6 expression in polarized RACK1-deficient cells than in the WT controls (Fig 6B). As anticipated, niclosamide treatment led to a clear reduction of pSTAT3 (Tyr^705^) in polarized RACK1-sufficient (WT) cells, more importantly, when the level of pSTAT3 was reduced to that of RACK1-deficient cells, the discrepancy in Bcl-6 expression vanished entirely (Fig 6A and 6B), indicating that the impaired STAT3 activity caused by RACK1 deficiency considerably explains the defective Bcl-6 expression during Tfh differentiation.

Furthermore, to investigate whether the defective STAT3 activity and Bcl-6 expression could be rectified by enforced expression of RACK1 in RACK1-deficient CD4^+^ T cells, we constructed a retroviral vector expressing RACK1 (MSCV-*Rack1*-IRES-GFP, OE-Rack1) and transduced pre-activated CD4^+^ T cells with it, or with an empty vector expressing GFP alone (MSCV-IRES-GFP, EV), then the transduced (GFP^+^) cells were sorted and cultured under the Tfh-like skewing condition. IB analysis confirmed RACK1 overexpression in RACK1-deficient cells incorporated with *Rack1* (Fig 6C). Again, the pSTAT3 (Tyr^705^) MFI was substantially lower in RACK-deficient cells than in their WT counterparts. Notably, enforced expression of RACK1 led to largely rescued phosphorylation of STAT3 in the Tfh-polarized cells deficient for RACK1 (Fig 6D). As a result, Bcl-6 expression was also substantially increased in RACK1-deficient cells transduced with the OE-Rack1 vector, the level was elevated nearly to that in the EV-transduced WT cells (Fig 6E). These data suggest that RACK1 deficiency is responsible for the defective STAT3 activity and subsequent Bcl-6 expression during Tfh differentiation.

### RACK1 promotes STAT3 activation and Bcl-6 expression by stabilizing STAT3 protein in CD4^+^ T cells

**Fig 7:**
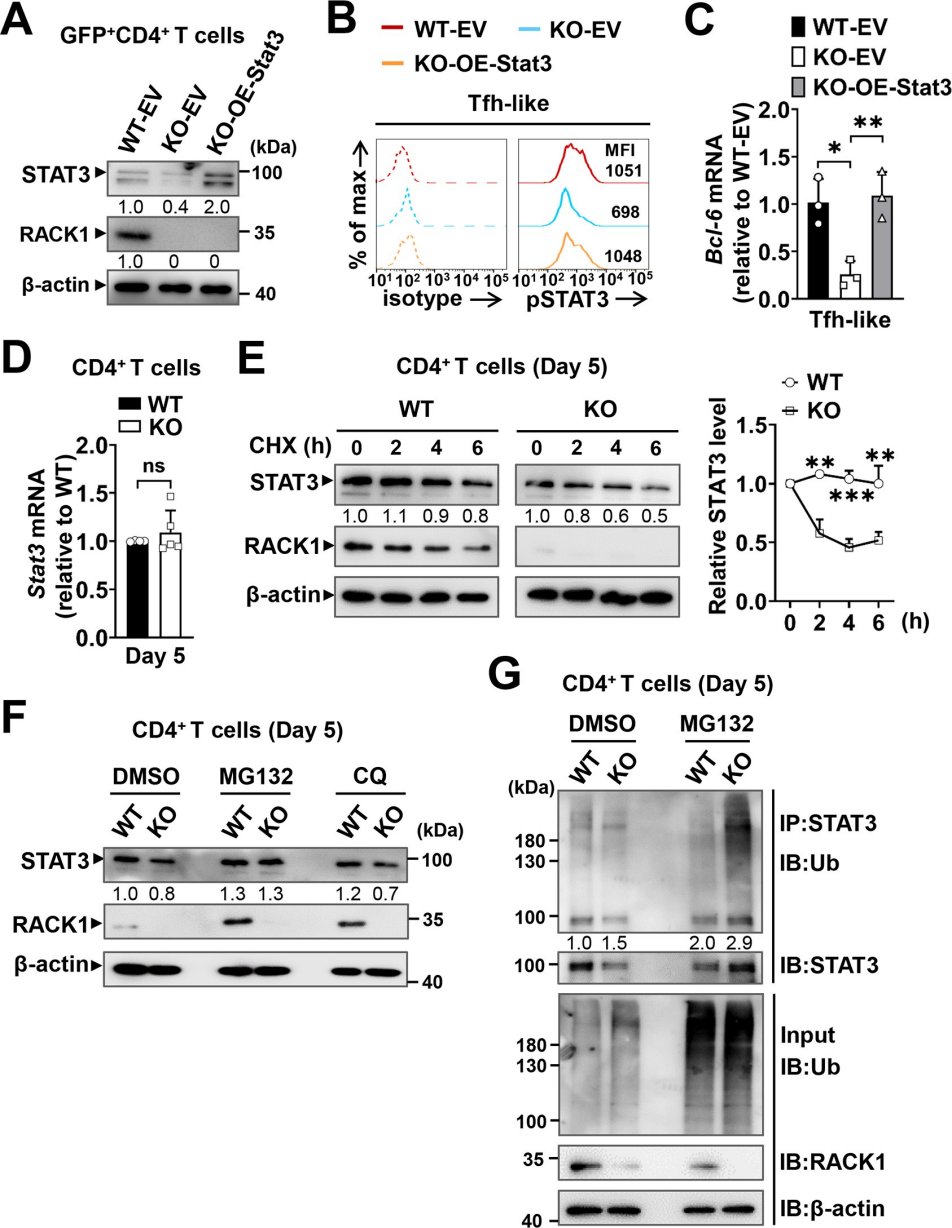
RACK1 promotes STAT3 activation and Bcl-6 expression by stabilizing STAT3 protein in CD4^+^ T cells. (A-C) Naïve CD4^+^ T cells from WT and RACK1 KO mice were transduced with the STAT3 overexpression retroviral vector (OE-Stat3) or GFP-expressing alone empty vector (EV), the transduced (GFP^+^) cells were then polarized under the Tfh-like condition for 3 days. (A) IB analysis of STAT3 protein levels in the retrovirally transduced GFP^+^CD4^+^ T cells. (B) Rrepresentative histograms showing pSTAT3 (Tyr^705^) in Tfh-like differentiated cells (48 h) transduced with the OE-Stat3 or EV vectors, the dash lines depict isotype IgG staining controls. Numbers indicate MFI of pSTAT3 in gated populations. (C) Quantitative RT-PCR for Bcl-6 expression in Tfh-like differentiated cells (n = 3/group). (D) Quantitative RT-PCR for Stat3 mRNA in splenic CD4^+^ T cells from WT and RACK1 KO mice at day 5 p.i. (n = 4-5/group). (E) Left, splenic CD4^+^ T cells from day 5-infected WT and RACK1 KO mice were treated with 10 μg/ml cycloheximide (CHX) for 0, 2, 4, 6 h, then subjected to IB analysis of STAT3, numbers indicate densitometry of the bands normalized to β-actin, relative to untreated cells (0 h). Right, summary graph showing quantification of relative STAT3 protein levels conducted by three independent experiments. (F) IB analysis of STAT3 in CD4^+^ T cells from day 5-infected WT and RACK1 KO mice treated with 20 μM MG132, 100 μM chloroquine (CQ), or vehicle (DMSO) for 8 h, respectively. Numbers showing band density normalized to β-actin, relative to WT cells treated with DMSO. (G) Splenic CD4^+^ T cells from day 5-infected WT and RACK1 KO mice were treated with 20 μM MG132 or DMSO for 8 h, endogenous ubiquitination of STAT3 was analyzed by IP with anti-STAT3 antibody, followed by IB with anti-ubiquitin (Ub) antibody. Numbers indicate densitometry of ubiquitinated-STAT3 normalized to the total amount of STAT3 in the immunoprecipitates. Data are representative of two or three independent experiments. *P<0.05, **P<0.01, ***P<0.001 and ns, not significant by one-way ANOVA with Tukey’s test (C), Student’s *t* test (D), two-way ANOVA with Bonferroni’s test (E).

The simultaneously decreased STAT3 and pSTAT3 (Tyr^705^) levels in RACK1-deficient CD4^+^ T cells (Fig 5) prompted us to speculate that the defective STAT3 activity might be attributable to STAT3 down-regulation caused by RACK1 deficiency. To test this possibility, naïve CD4^+^T cells from RACK1 KO mice were transduced with the retroviral vector expressing STAT3 (MSCV-*Stat3*-IRES-GFP, OE-Stat3) during their differentiation into Tfh-like cells, similar to overexpression of RACK1, cells from WT and KO mice transduced with the MSCV-IRES-GFP empty vector (EV) were served as controls. As expected, overexpression of STAT3 considerably upregulated the pSTAT3 level in Tfh-like cells lacking RACK1 (Fig 7A and 7B). Furthermore, Bcl-6 expression in these cells was greatly increased, almost equivalent to the level in WT cells transduced with EV (Fig 7C). Thus, enforced expression of STAT3 could reverse the defects of RACK1 deficiency, suggesting that RACK1 might play a key role in sustaining proper STAT3 protein level and, in consequence, promoting optimal STAT3 activity and Bcl-6 induction.

Then we sought to dissect how RACK1 regulates the protein amount of STAT3 in CD4^+^ T cells. The comparable *Stat3* mRNA levels in CD4^+^ T cells from day 5-infected WT and RACK1 KO mice suggested that the effect was not due to regulation at the transcript level (Fig 7D). Given that RACK1 modulates the degradation of several signaling molecules through either the proteasome- or lysosome-dependent pathways [62,63], our previous studies have also disclosed that RACK1 regulates the ubiquitination and stability of histone deacetylase 1 (HDAC1) during cerebellum development and fulminant hepatitis progression [64,65], we therefore asked if RACK1 exerted a similar effect on STAT3 stability in CD4^+^ T cells. Herein, we evaluated the half-life of STAT3 in CD4^+^ T cells after *P*. *yoelii* infection. To exclude the potential impact of RACK1 on protein synthesis, CD4^+^ T cells from day 5-infected WT and RACK1 KO mice were treated with the translational repressor, cycloheximide (CHX) for indicated periods. As expected, ablation of RACK1 accelerated the degradation of endogenous STAT3 protein (Fig 7E), confirming the functional role of RACK1 in maintaining STAT3 stability in CD4^+^ T cells. To further assess which pathway is responsible for this effect, CD4^+^ T cells from day 5-infected mice were treated with the proteasome inhibitor MG132, the lysosome inhibitor chloroquine (CQ), or DMSO, respectively. Notably, MG132 treatment led to a remarkable increase in STAT3 protein both in WT and RACK1-deficient CD4^+^ T cells, more importantly, the level in RACK1-deficient cells was elevated even comparable to that in their WT counterparts (Fig 7F). However, CQ treatment did not alter the abundance of STAT3 protein in RACK1-deficient cells, even though the level was slightly increased in the same treated WT controls (Fig 7F). Consistent with this, in the presence of MG132, a markedly enhanced ubiquitination of STAT3 was found in RACK1-deficient CD4^+^T cells compared with that in CD4^+^ T cells from WT mice (Fig 7G). Together, RACK1 may function to stabilize STAT3 protein probably via suppressing the ubiquitin-proteasomal degradation process in CD4^+^ T cells.

### RACK1 attenuates STAT3 interaction with Wwp2 and Itch in CD4^+^ T cells during *P*. *yoelii* infection

**Fig 8:**
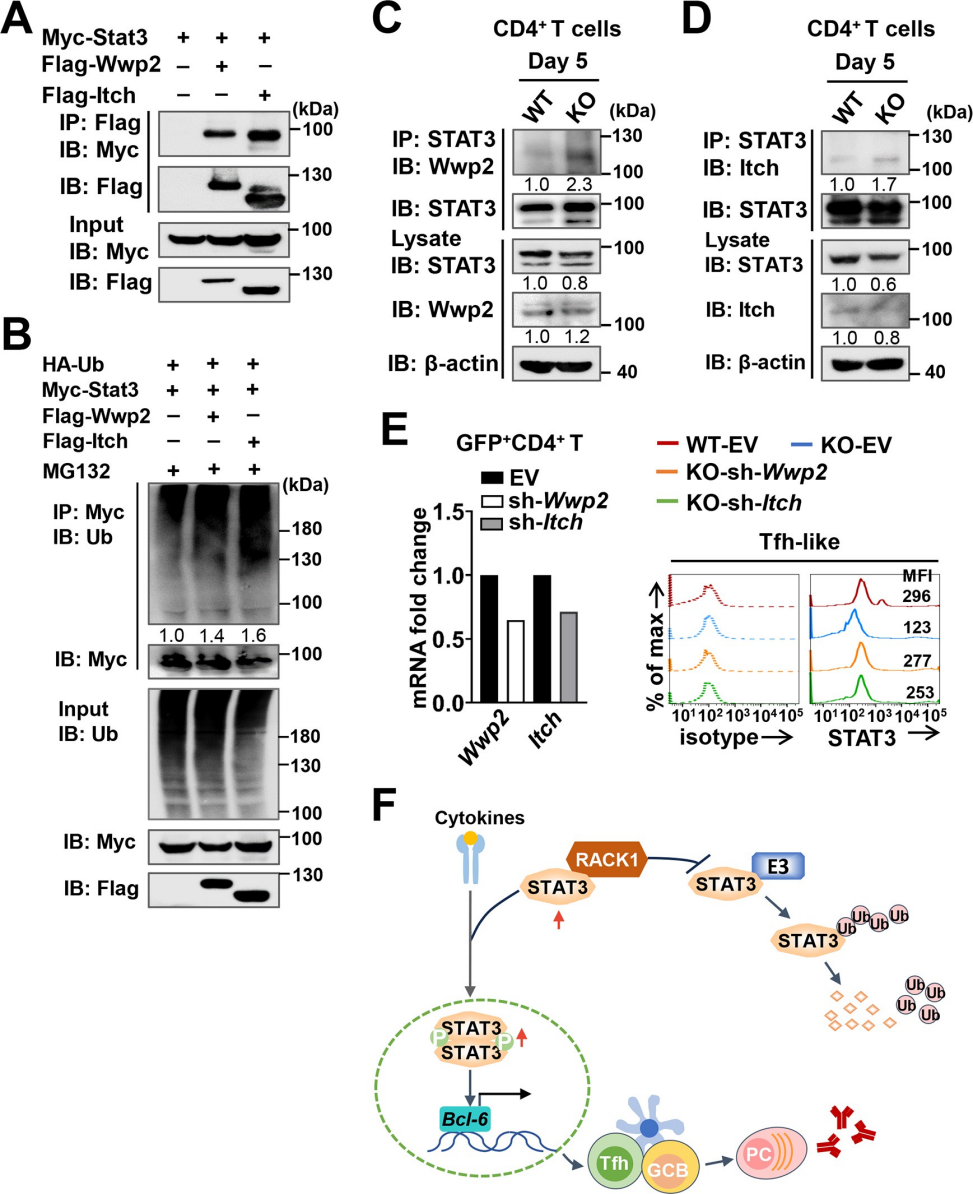
Wwp2 and Itch are potential ubiquitin ligases implicated in RACK1-attenuated STAT3 degradation. (A) The interactions between STAT3 and Wwp2 (or Itch) in 293T cells transfected with plasmids expressing Myc-tagged *Stat3* and Flag-tagged *Wwp2* (or *Itch*) were detected by IP with an anti-Flag antibody, followed by IB with an anti-Myc antibody. Lysates without IP were prepared for IB of plasmid expression. (B) Ubiquitination of STAT3 in 293T cells transfected with Myc-tagged *Stat3* and HA-tagged Ubiquitin (Ub), with or without Flag-tagged *Wwp2* (or *Itch*) co-transfection was analyzed by IP with an anti-Myc antibody, followed by IB with an Ubiquitin antibody. MG132 was added for the last 8 h before cell collection. Numbers indicate densitometry of ubiquitinated-Myc-STAT3 normalized to the total amount of Myc-STAT3 in the immunoprecipitates. (C-D) Endogenous interaction between STAT3 and Wwp2 (or Itch) in CD4^+^ T cells from WT and RACK1 KO mice at day 5 p.i. was determined by IP with anti-STAT3 antibody, followed by IB with anti-Wwp2 (or anti-Itch) antibodies. Lysates without IP were prepared for IB analysis of STAT3 and Wwp2 (or Itch) expression. (E) CD4^+^ T cells from WT and RACK1 KO mice were transduced with the retroviral vectors expressing shRNA targeted *Wwp2* (sh-*Wwp2*), *Itch* (sh-*Itch*), or GFP alone (EV), the GFP^+^ cells were then polarized under the Tfh-like condition. Left, quantitative RT-PCR for *Wwp2* (or *Itch*) mRNA in GFP^+^CD4^+^ T cells. Right, representative histograms showing the MFI of STAT3 in Tfh-like differentiated cells (24 h) retrovirally transduced with sh-*Wwp2*, sh-*Itch*, or EV, the dash lines depict isotype IgG staining controls. (F) Schematic diagram showing the mechanism of RACK1-regulated Tfh differentiation and function. RACK1 may work to prevent the attachment of certain E3 ligases to STAT3 and subsequent degradation of STAT3 by the ubiquitin-proteasome system. This leads to enhanced stability and phosphorylation activity of STAT3 in CD4^+^ T cells, which ultimately promotes Bcl-6 induction, Tfh development, and generation of parasite-specific antibodies during *P*. *yoelii* infection. Data in (A-E) are representative of two independent experiments with similar results.

Finally, to comprehensively address how RACK1 attenuates ubiquitin-dependent degradation of STAT3, we attempted to search for possible E3 ubiquitin ligases that target STAT3 for degradation. For this purpose, we re-examined the transcriptome profile of our previous work (https://www.ncbi.nlm.nih.gov/geo/query/acc.cgi?acc=GSE111066) and found that in activated CD4^+^ T cells of *P*. *yoelii* 17XNL-infected mice, some highly expressed E3 ligases, such as Wwp2 and Itch were predicted as candidate E3 ligases of STAT3 (UbiBrowser 2.0), we therefore asked if these molecules provide a possible link between RACK1 and STAT3 degradation. Firstly, we analyzed the interaction between STAT3 and Wwp2 (or Itch) in HEK-293T cells. As expected, specific conjugation of Wwp2 with STAT3 was confirmed in 293T cells co-transfected with Flag-tagged *Wwp2* and Myc-tagged *Stat3* plasmids (Fig 8A). Moreover, in the presence of MG132, overexpression of Wwp2 enhanced the ubiquitination of STAT3 (Fig 8B). Similar results were obtained in 293T cells transfected with plasmid encoding Flag-tagged *Itch* (Fig 8A and 8B), suggesting that Wwp2 and Itch might be specific ubiquitin ligases of STAT3. On this basis, we then investigated the relationship between STAT3 and these E3 ligases in CD4^+^ T cells. As expected, Co-IP analysis confirmed the endogenous conjugation of STAT3 with Wwp2 (or Itch) in splenic CD4^+^ T cells from *P*. *yoelii*-infected mice (Fig 8C and 8D). Moreover, loss of RACK1 enhanced the interaction of STAT3 with these E3 ligases at day 5 p.i. (Fig 8C and 8D), indicating that Wwp2 or Itch might function as potential E3 ligases involved in RACK1-attenuated STAT3 degradation in CD4^+^ T cells. To test this, we constructed retrovirus encoding short hairpin RNA (shRNA) targeted Wwp2 or Itch, and used the pMKO.1-GFP mediated knockdown system to assess whether knockdown of such potential E3 ligases could increase the protein amount of STAT3 during Tfh-like cell differentiation. The shRNA silencing efficiency in retrovirally transduced GFP^+^CD4^+^T cells was confirmed by quantitative RT-PCR (Fig 8E). As anticipated, STAT3 protein levels were largely restored in Tfh-polarized RACK1-deficient cells that knockdown of Wwp2 (or Itch) (Fig 8E). Altogether, we propose that RACK1 might play a role in suppressing the interaction between STAT3 and potential E3 ligases, such as Wwp2 and Itch, thus favoring the maintenance of STAT3 stability and optimal STAT3 activity during the Tfh differentiation process. Consequently, RACK1 enhances Tfh cell expansion and subsequent production of anti-parasite antibodies in response to *P*. *yoelii* infection. (Fig 8F).

## Discussion

As an adaptor protein, RACK1 has been reported to recruit specific signaling elements and participate in diverse cellular functionalities [40–45]. However, the impact on host protective immunity against infectious diseases is yet to be investigated. The results presented here highlight the significance of RACK1 as a key regulator of CD4^+^ T cell-mediated immunity to *Plasmodium* infection. Particularly, we identified a novel role of RACK1 in orchestrating the Tfh polarization program and subsequent germinal center reaction, which contributes to the development of functional antibodies and ultimately immune resistance to non-lethal blood-stage *P*. *yoelii* 17XNL infection.

Our prior research has shown that *Gnb2l1*^fl/fl;CD4-Cre^ or *Gnb2l1*^fl/fl;Lck-Cre^ mice exhibited a noticeable decrease in peripheral CD4^+^ and CD8^+^ T cell proliferation, along with an increase in cell apoptosis, which could be explained by impaired autophagy in T cells with RACK1 deficiency [50]. Accordingly, we assumed this homeostatic defect might influence the magnitude of CD4^+^ T cell responses to *Plasmodium* infection. Here, the generation of the *Gnb2l1*^fl/fl^CD4CreER^T2^ mouse model allowed us to overcome this limitation. In this model, specific deletion of RACK1 in CD4^+^ T cells was achieved through a tamoxifen-inducible approach (Fig 1). As expected, initial activation and numbers of splenic CD4^+^ T cells were not affected under steady-state conditions. As such, the observed discrepancy in anti-*Plasmodium* immunity was entirely due to the effects of RACK1 on CD4^+^ T cell responses after parasite challenge. Consistent with the finding of TCR ligation *in vitro* [50], in this study, we confirmed a prominent role of RACK1 in regulating *P*. *yoelii* 17XNL-elicited CD4^+^ T cell activation and proliferation. It should be noticed, however, that no detectable difference in cell viability was found in RACK1-deficient CD4^+^ T cells compared to their WT counterparts, one possibility may be that compensatory signals or molecules also participate in regulating CD4^+^ T cell survival *in vivo*. Therefore, RACK1 is mainly required for CD4^+^ T cell expansion during the early phase of *P*. *yoelii* infection.

One predominant CD4^+^ helper T cell subset that emerges following *Plasmodium* infection is Th1, the major source of IFN-γ, which we and others have revealed its importance in limiting parasite replication early after blood-stage *P*. *yoelii* and *P*. *chabaudi* infection [19,20,53]. In addition, studies have also elucidated the vital role of Treg cells in suppressing excessive proinflammatory responses and resolving *P*. *yoelii* infection [53]. Here, IFN-γ secretion by CD4^+^ T cells was assessed by restimulating parasite-primed cells *ex vivo*. Although the PMA/ionomycin stimulation approach may not accurately reflect Th1 cell response elicited by *P*. *yoelii*, the normal frequency of IFN-γ-producing cells suggests the potential of CD4^+^ T cells to differentiate into the Th1 lineage is not impaired by the absence of RACK1. Instead, the decreased cell number in RACK1 KO mice is largely attributed to the defective expansion of CD4^+^ T cells in response to *P*. *yoelii*. A similar phenomenon was found in the Th17 lineage, which might also be implicated in controlling blood-stage malaria [54]. Meanwhile, although the proportions of regulatory T cell subsets, such as nTreg and Tfr were noted to be increased in the absence of RACK1, the absolute numbers were still lower (nTreg) or comparable (Tfr) to those in WT mice. Likely, the simultaneously reduced nTreg and IFN-γ (or IL-17A)-secreting CD4^+^ T cells may considerably explain the comparable parasitemia in WT versus RACK1 KO mice during the early phase of infection. Moreover, as the elevated proportions of nTreg and Tfr cells could not be able to compensate for the remarkably decreased Tfh proportion in the CD4^+^ T cell compartment, it is reasonable to speculate that other potential cell subsets might also be affected by RACK1 deficiency, further efforts will be needed to elucidate this issue.

Another effector CD4^+^ T cell subset that contributes to parasite clearance is Tfh, which has been well-documented for its capability of supporting B cell-mediated humoral immunity [27,30]. Here, we provide *in vivo* evidence that loss of RACK1 led to impaired Tfh cell development and function during *P*. *yoelii* 17XNL infection. The defect was not only due to impairment of the proliferative capacity of CD4^+^ T cells, but also the attenuated propensity to differentiate into the Tfh lineage, as evidenced by severely decreased proportions of Tfh cells and GC Tfh cells, along with defective transcription of Tfh signature genes such as *Bcl-6*, *Cxcr5*, *Pdcd-1* and *Il-21* in RACK1-deficient CD4^+^ T cells early after infection (Fig 3). The impact on Tfh cell proportions and numbers persists until the later phase of the parasite challenge. Consequently, the GC reaction and parasite-specific antibody response were dramatically compromised during this *P*. *yoelii* infection.

As a key regulator, Bcl-6 has a central role in orchestrating Tfh cell differentiation, either via upregulating molecules that are critical for Tfh migration and function, or repressing the transcriptional repressor Blimp1 and those factors direct alternative Th lineages [33,66,67]. Here, we describe a previously unrecognized role for RACK1 in Bcl-6 expression, which considerably explains the abnormal Tfh phenotype caused by RACK1 deficiency. However, it could not rule out the possibility that RACK1 is also involved in regulating other Tfh-related molecules, future studies will be required to fully elucidate whether additional pathways act synergistically to control Tfh development during *Plasmodium* infection. Mechanistically, the effect of RACK1 on Bcl-6 expression seems not to be associated with the Blimp1/Bcl-6 axis, and the potent regulators such as TCF-1 and SAP, but probably relies on STAT3 activity. In common with other STAT proteins, STAT3 is activated by cytokine signal-triggered phosphorylation on the tyrosine residual. IL-6, IL-21 and IL-27, the dominant drivers of the Tfh lineage, act predominantly on STAT3-dependent mechanisms [27,68,69]. As such, STAT3 functions as the most important inducer of Bcl-6. STAT1, another transcription factor of the STAT family, has been reported to participate in type I IFN-induced Bcl-6 expression, or cooperate with STAT3 to mediate IL-6-derived Tfh differentiation in some circumstances [37,70]. Previous work also reported specific interaction between RACK1 and STAT1, and the contribution of RACK1 to STAT1 activation initiated by type I IFNs [45]. Here, we describe a novel relationship between RACK1 and STAT3 in CD4^+^ T cells upon *P*. *yoelii* infection, this finding expands the importance of RACK1 in activating different STATs, and highlights the dominant factors required for initiating Tfh differentiation in various models. The impaired tyrosine phosphorylation coincided with substantially decreased STAT3 protein in *P*. *yoelii*-primed CD4^+^ T cells and the *in vitro* generated Tfh-like cells emphasized a requirement for RACK1 in initiating optimal STAT3 activity during Tfh differentiation, which was further confirmed by enforced expression of RACK1 in RACK1-deficient CD4^+^ T cells. In addition, a slight decrease in STAT1 phosphorylation was noted in the absence of RACK1, even though the protein level was unaltered in CD4^+^ T cells upon *P*. *yoelii* infection. It could not be ruled out that STAT1 might function redundantly or cooperate with STAT3 to allow optimal induction of Bcl-6 during *Plasmodium* infection. However, STAT3 may represent the major factor in the RACK1-regulated Tfh differentiation process. This is strongly supported by the finding that enforced expression of STAT3 in RACK1-deficient CD4^+^ T cells almost entirely rescued the defects in STAT3 activity and Bcl-6 expression during Tfh-like cell polarization. STAT5, an important negative regulator of the Tfh program, was not found to be affected by the absence of RACK1, this is consistent with the transcript data showing that Blimp1 expression was comparable in WT versus RACK1-deficient CD4^+^ T cells upon *P*. *yoelii* infection. Furthermore, the impaired STAT3 phosphorylation induced by RACK1 deficiency seems not correlated with a defect in the potent kinase Jak1 expression [71], as Jak1 levels were not significantly different in CD4^+^ T cells from WT and RACK1 KO mice at day 5 p.i. (S7A Fig). By contrast, further mechanistic analyses suggested that RACK1 promotes STAT3 activation predominantly via its functionality in maintaining higher STAT3 level. Indeed, decreased Ser^727^ phosphorylation of STAT3 was also found in RACK1-deficient CD4^+^ T cells at day 5 p.i. (S7B Fig), which further supports the conclusion that the diminished tyrosine phosphorylation of STAT3 is largely attributable to down-regulation of STAT3 induced by RACK1 deficiency during the Tfh skewing process.

We additionally confirmed an increase in ubiquitinylated STAT3 as well as the abundance of STAT3 protein in RACK1-deficient CD4^+^ T cells by MG132 treatment, thus supporting the view that RACK1 maintains STAT3 stability predominantly via the ubiquitin-proteasome pathway. For the molecular mechanism, we searched for possible ubiquitin ligases mediating STAT3 degradation. Previous studies have reported implications of MDM2 and COP1 that targeted STAT3 for degradation in tumorigenesis [72,73], and involvement of MDM2 in RACK1-abrogated HDAC1 degradation during the pathogenesis of fulminant hepatitis [65]. Unexpectedly, we could not detect endogenous interaction between STAT3 and MDM2 (or COP1) in *P*. *yoelii-*primed CD4^+^T cells. By contrast, we identified that Wwp2 and Itch might be potential E3 ligases involved in RACK1-attenuated STAT3 ubiquitination and degradation. This discrepancy may arise from the different substrates and distinct regulatory mechanisms of RACK1 in various cell types. Both Wwp2 and Itch belong to the HECT-type ubiquitin ligases, Wwp2 has been recognized for its regulation of STAT3 acetylation and phosphorylation in vascular smooth muscle cells [74], and Itch has been reported to contribute to Tfh differentiation via targeting the negative regulator Foxo1[75]. However, their functional relationship with STAT3 in T cells remains elusive. Here, we elucidated the potential role of these E3 ligases during Tfh differentiation. By the shRNA-mediated knockdown approach, the similarly rescued STAT3 protein levels in Tfh-polarized cells suggested that Wwp2 and Itch may function synergistically in the RACK1-attenuated STAT3 degradation process, future studies are required to explore details of the molecular mechanisms. Meanwhile, concerning the wide-ranging effects of RACK1 on multiple signals and factors, additional E3 ligases might also be implicated in this process. Comprehensive investigations will facilitate the identification of targets to engender optimal CD4^+^ T cell responses and stronger humoral immunity to *Plasmodium* infection.

In summary, we have elucidated the biological effects of RACK1 on CD4^+^ T cell responses and subsequent humoral immunity in a murine malaria model. The findings provide mechanistic insight into posttranslational regulation of STAT3 in Tfh cell development and function, which broadens our understanding of the molecular regulation of effector CD4^+^ T cell responses in blood-stage malaria. However, there are limitations in deducing the regulatory role of RACK1 and its corresponding pathways in humans. Future efforts on this issue would advance our knowledge of immune regulation in human malaria and facilitate the development of better control strategies.

## Materials and methods

### Ethical statements

All animal experiments were performed in strict accordance with the Guide for the Care and Use of Laboratory Animals in Research of the People’s Republic of China. Experimental procedures were approved by the Institutional animal care and use committee of Beijing Institute of Basic Medical Sciences (Permit number: IACUC-DWZX-2021-567). All efforts were made to minimize suffering.

### Mice

Mice harboring *loxP*-flanked *Gnb2l1* alleles (*Gnb2l1*^fl/fl^) on a C57BL/6 background were generated as previously described [50]. Mice expressing tamoxifen-inducible CreER^T2^ recombinase driven by the *Cd4* promoter were purchased from The Jackson Laboratory (Bar Harbor, ME, USA). *Gnb2l1*^fl/fl^ mice were crossed with CD4CreER^T2^ mice to generate *Gnb2l1*^fl/fl^CD4CreER^T2^ mice, in which deletion of *Gnb2l1* (encoding RACK1) in the CD4^+^ T cell compartment was achieved by intraperitoneal (i.p.) injection with 2 mg tamoxifen (T5648, Sigma-Aldrich) for consecutive 5 days. The same treated *Gnb2l1*^fl/fl^ littermates were served as controls. All mice were maintained under specific pathogen-free conditions and used between 6 to 8 weeks of age.

### Antibodies for flow cytometry and immunoprecipitation/immunoblotting assay

The antibodies and reagents utilized for flow cytometry (FC), immunoprecipitation (IP) and immunoblotting (IB) in this study are listed in S1 Table.

### *Plasmodium* infection

The cloned line of *P*. *yoelii* 17XNL was originally obtained from Dr. Weiqing Pan (Navy Medical University, Shanghai, China). Parasites were maintained as frozen stocks and passaged through donor mice before infecting experimental mice. Blood-stage *Plasmodium* infection was initiated by i.p. injection of 3×10^4^ parasitized erythrocytes. Parasitemia was assessed by Giemsa-stained thin smears of tail blood. Mortality was monitored daily throughout the course of infection.

### *P*. *yoelii*-specific IgG ELISA

Serum samples were collected at indicated time points of infection. Parasite-specific IgG was detected by coating 96-well microtiter plates with crude plasmodial antigen of *P*. *yoelii* 17XNL, which was prepared as described previously [53]. The coated well was blocked with 1%BSA in PBST (PBS containing 0.1% Tween-20) for 1 h at room temperature, followed by sequential incubation with pre-diluted (1:100) serum samples for 2 h and HRP-conjugated goat anti-mouse IgG (Santa Cruz biotechnology) for 1 h at room temperature. 3,3’,5,5’-tetramethylbenzidine (TMB, Biolegend) was used as substrate and absorbance was read at 450 nm. Data were presented as OD values.

### Flow cytometric analysis

Splenocytes isolated from naive or infected mice were Fc blocked with anti-mouse CD16/32 and surface stained with appropriate combinations of fluorochrome-conjugated antibodies for 30 min at 4°C. CXCR5 staining was performed by incubation with biotinylated anti-CXCR5 (BD Pharmingen) for 1 h at 4°C, followed by incubation with PE-labeled streptavidin (BD Pharmingen) for 30 min at 4°C. For cell apoptosis, surface-stained splenocytes were incubated in 1×binding buffer with Annexin V followed by staining with 7-AAD (eBioscience). For intracellular cytokine staining, cells were stimulated with 50 ng/ml Phorbol-12-myristate-13-acetate (PMA, Sigma-Aldrich), 500 ng/ml ionomycin (Sigma-Aldrich) and brefeldin A (1:1000, eBioscience) at 37°C for 5 h. After surface staining, the cells were fixed with 2% paraformaldehyde and permeabilized with 0.1% saponin (Sigma-Aldrich), followed by staining with Brilliant Violet 650-conjugated anti-IFN-γ and PerCP/Cy5.5-conjugated anti-IL-17A (Biolegend). Intracellular transcription factor staining was performed using Foxp3/transcription factor staining buffer set (eBioscience), APC-conjugated anti-Foxp3 (eBioscience), primary antibodies against Bcl-6 (BD Pharmingen) and STAT3 (Proteintech), and appropriate secondary antibodies conjugated with FITC (ABclonal). For phosflow analysis of pSTAT3, cells were fixed with BD Phosflow lyse/Fix buffer at 37°C for 10 min, permeabilized with BD Phosflow Perm Buffer II for 30 min on ice, followed by incubation with Alex Fluor 647-conjugated anti-pSTAT3 or isotype controls (BD Biosciences) at room temperature for 30–60 min, according to the manufacturer’s protocol. All flow cytometric data were acquired on a FACS Celesta (BD Biosciences) and analyzed with FlowJo V10 software (TreeStar).

### Immunofluorescence and confocal microscopy

Freshly isolated spleens were snap-frozen in Tissue-Tek OCT compound (Sakura Finetek), 6–8 μm serial sections were fixed with ice-cold acetone for 10 min at -20°C. The sections were blocked with 3% BSA at room temperature for 30 min, followed by staining with a mixture of Alexa Fluor 594-conjugated rat anti-mouse CD3 (17A2, Biolegend), Alexa Fluor 488-conjugated rat anti-mouse B220 (RA3-6B2, Biolegend) and Alexa Fluor 647-conjugated rat anti-mouse GL7 (GL7, Biolegend) at 4°C overnight. Then the slides were washed with PBST three times and mounted with anti-fade mounting medium (Beyotime Biotechnology). The stained sections were scanned on an Olympus SLIDEVIEW VS200 slide scanner and analyzed with Olyvia software (Olympus).

### RNA extraction and quantitative RT-PCR

Total RNA from primary CD4^+^ T or *in vitro* cultured cells was extracted with Trizol reagent (Life Technologies) and converted to cDNA using ABScript II RT Master Mix (Abclonal) according to the recommended protocol. Quantitative Real-Time PCR was performed using FastStart Essential DNA Green Master Mix (Roche) and the LightCycler 480 Real-Time PCR system (Roche). Relative gene expression was normalized to the *β-actin* internal control and calculated using the 2^-ΔΔCt^ method. The primer sequences used for RT-PCR are listed in S2 Table.

### CD4^+^ T cell enrichment and culture

CD4^+^ T cells were enriched from spleens of naïve or *P*. *yoelii*-infected mice using CD4^+^ T cell isolation kit or CD4 microbeads (Miltenyi Biotec), with the purity routinely >95%. Following isolation, cells were cultured in RPMI1640 medium supplemented with 10% heat-inactivated fetal bovine serum, 100U/ml penicillin, 100 μg/ml streptomycin, 2 mM L-glutamine and 50 μM β-mercaptoethanol (all from Gibco). For STAT3 protein stability, 10 μg/ml cycloheximide (CHX, Sigma-Aldrich) or vehicle control (DMSO, Sigma-Aldrich) was added to the culture media at 0, 2, 4, 6, and 8 h before cell collection. To analyze the pathway mediating STAT3 degradation, 20 μM MG132 (absin), 100 μM chloroquine (Sigma-Aldrich), or vehicle (DMSO) was added to the media 8 h before cell collection. For *in vitro* Tfh-like cell differentiation, FACSAria III sorted naïve CD62L^hi^CD44^lo^CD25^-^CD4^+^ T cells or retroviral transduced GFP^+^CD4^+^ T cells were activated with PMA/ionomycin and cultured under the condition with 10 μg/ml anti-IFN-γ (XMG1.2, Invitrogen), 10 μg/ml anti-IL-4 (11B11, Invitrogen), 10 μg/ml anti-IL-2 (JES6-1A12, Invitrogen), 20 μg/ml anti-TGF-β (1D11, BioXCell), 20 ng/ml murine recombinant IL-6 (Miltenyi Biotech) and 50 ng/ml IL-21 (R&D Systems) for indicated periods, according to previously published procedure [39]. In some experiments, 1μM STAT3 inhibitor niclosamide (Selleckchem) or vehicle (DMSO) was added to the culture media on day 2 of Tfh-like polarization, as previously described [61].

### HEK-293T cell culture and plasmid transfection

Plasmids encoding Myc-tagged *Stat3* and HA tagged-Ubiquitin were purchased from Vigene Biosciences, Flag-tagged *Wwp2* and Flag-tagged *Itch* were kindly provided by Dr. Lingqiang Zhang (Institute of Lifeomics, Beijing, China). HEK-293T cells were cultured in Dulbecco’s Modified Eagle medium (Gibco) supplemented with 10% heat-inactivated fetal bovine serum, 100U/ml penicillin, and 100 μg/ml streptomycin. Plasmid transfection was performed using jetPRIME reagent (Polyplus), according to the manufacturer’s protocol. To analyze the ubiquitination of STAT3, MG132 was added for the last 8 hours of culture, as described above. The resulting samples were harvested and processed for Co-IP or IB analysis.

### Plasmid construction and retroviral transduction in CD4^+^ T cells

MSCV-IRES-GFP and pMKO.1GFP retroviral vectors were obtained from Addgene (plasmids #20672, #10676), the pCL-Eco packaging vector was provided by Dr. Lin Sun (Renji Hospital, Shanghai, China). For gene overexpression, total RNA of CD4^+^ T cells from day 5-infected *Gnb2l1*^fl/fl^ (WT) mice was isolated, reverse transcribed using RevertAid First Strand cDNA Synthesis Kit (Thermo Fisher Scientific), and PCR amplified using Q5 High-Fidelity DNA Polymerase (M0491, NEB). MSCV-IRES-GFP vector was digested by EcoRI/XhoI restriction enzymes (R3101 and R0146, NEB), the coding sequences of *Gnb2l1* or *Stat3* were cloned into MSCV-IRES-GFP using 2×seamless cloning Mix (CL117-01, Biomed), respectively. For gene knockdown, shRNA for *Wwp2* and *Itch* were synthesized, annealed, and ligated with the EcoRI/XhoI digested pMKO.1 backbone using T4 DNA ligase (M0202, NEB). The resulting constructs or empty vector and pCL-Eco were transfected into HEK-293T cells as described [39], and cell-free supernatants containing viral particles were harvested 48 h post-transfection. CD4^+^ T cells were pre-activated with CD3/CD28 Dynabeads (Gibco) for 24 h and spin infected with the retrovirus in the presence of 8 μg/ml polybrene (Sigma-Aldrich) at 32°C for 90 min. After incubating at 37°C for 24 h, the transduced GFP^+^CD4^+^ T cells were sorted for gene expression analysis or *in vitro* differentiation assay. The shRNA targeting sequences are listed in S2 Table.

### Immunoblotting (IB) and immunoprecipitation (IP) analysis

Cells harvested for IB were washed with ice-cold PBS and lysed with RIPA buffer (50 mM Tris-HCl pH7.5, 150 mM NaCl, 1% NP-40, 0.1% SDS) supplemented with the protease/phosphatase inhibitor mixture. Equal amounts of protein were separated with 10–15% polyacrylamide gels, electrotransferred onto polyvinylidene difluoride membranes (Millipore), and blotted with a recommended concentration of primary antibodies at 4°C overnight, then washed and incubated with corresponding Horseradish peroxidase (HRP)-conjugated secondary antibodies at room temperature for 1 h. Immunoreactive bands were visualized by the ECL Chemiluminescence Kit (Thermo Fisher Scientific). For the Co-IP assay, cells were homogenized with Co-IP lysis buffer (10 mM Tris-Cl pH7.5, 2 mM EDTA, 1% NP-40, 150 mM NaCl) in the presence of protease/phosphatase inhibitor mixture, followed by incubation with appropriate primary antibodies and Protein A/G-plus agarose (Santa Cruz biotechnology) at 4°C for 4 h. The resulting precipitates were washed with lysis washing buffer 5 times and subjected to IB analysis. Immunoblotting densitometry was analyzed using Image J software (National Institutes of Health).

### Silver staining and protein identification by mass spectrometry

CD4^+^ T cell lysates from naïve or day 5-infected mice were prepared using Co-IP lysis buffer, the RACK1-immunoprecipitated samples were subjected to SDS-PAGE, then fixed in 50% methanol and 10% acetic acid for 20 min, after rinsing with 20% ethanol for 10 min and washing with distilled water, the gel was agitated in 0.02% sodium-thiosulfate for 1 min, followed by washing and incubating in 0.1% silver nitrate for 20 min, then incubated in sodium carbonate and formaldehyde developing solution, and stopped with 5% acetic acid. Specific protein bands were cut and subjected to mass spectrometry using an AB Sciex 5600+ TripleTOF mass spectrometer (Concord, Ontario, Canada) interfaced to a NanoLC 425 system (Dublin, CA) as previously described [76]. Briefly, peptides were trapped on a NanoLC pre-column (Chromxp C18-LC-3μ m, Eksigent), eluted onto an analytical column (C18-CL-120, Eksigent) and separated at a flow rate of 300nL/min. Full-scan MS was performed in positive ion mode with a nano-ion spray voltage of 2.3 kv from 350 to 1500 (m/z), with up to 50 precursors selected for MS/MS (m/z 100–1500). The selection criteria for parent ions included an intensity greater than 150 counts/s, a charge state from +2 to +5, a mass tolerance of 50mDa and dynamic exclusion for 15 s. Ions were fragmented in the collision cell using rolling collision energy. Mass spectrometry data were submitted to search through the database SWISS-PROT (version 2016_10; 552,884 sequences; 197,760,918 residues) via a Mascot Daemon 2.4.1 server, *Mus musculus* was selected as specific species. The mass accuracy for parent ions was set as ± 30 ppm, and ± 0.2 Da was used for the fragment ion mass tolerance. Proteins were identified on the basis of three or more different peptides whose ion scores exceeded the threshold of *P* < 0.05, indicating identification at the 95% confidence level.

### Statistics

Data were analyzed using Prism 9 software (GraphPad) and expressed as mean±SD. Statistical analyses were performed using two-tailed Student’s *t* test or non-parametric Mann-Whitney test for two-group comparisons, and one-way ANOVA with Tukey’s test or two-way ANOVA with Bonferonni’s test for more than two-group comparisons. Kaplan-Meier curves of overall survival were compared using the log-rank test. A *P* value < 0.05 was considered to be statistically significant.

## Supporting information

S1 Fig CD4^+^ T cell-targeted RACK1 deficiency does not affect other T cell homeostasis prior to *P*. *yoelii* infection. (A) Representative dot plots and (B-C) bar graphs showing the proportions and numbers of CD8^+^ T (CD3^+^CD8^+^), NKT (CD3^+^NK1.1^+^), and γδ T (CD3^+^TCRγ/δ^+^) cells in the spleen of WT and RACK1 KO mice before *P*. *yoelii* 17XNL infection. (D) Representative dot plots and bar graph showing the proportions of naïve (CD62L^hi^CD44^lo^) CD8^+^ T cells in the spleen of WT and RACK1 KO mice before *P*. *yoelii* 17XNL infection. Data are pooled from two independent experiments with 3–6 mice/group and are shown as mean±SD. ns, not significant by Student’s *t* test (B, D) or Mann-Whitney test (C).
(TIF)

S2 Fig RACK1 is required for CD4^+^ T but not other T cell expansion after *P*. *yoelii* 17XNL infection. (A) Representative dot plots and bar graphs showing the proportions and numbers of CD4^+^ T cells in the spleen of WT and RACK1 KO mice at day 16 p.i. (B) Representative dot plots and bar graphs showing the proportions and numbers of CD8^+^ T cells, NKT cells, and γδ T cells in the spleen of WT and RACK1 KO mice at day 7 p.i. Data are pooled from two independent experiments with 5–6 mice/group and are shown as mean±SD. ns, not significant by Student’s *t* test.
(TIF)

S3 Fig RACK1 promotes global Tfh cell development without affecting Tfh viability and Tfr differentiation. (A) Gating strategy and frequencies of apoptotic (Annexin V^+^) Tfh cells in the spleen of WT and RACK1 KO mice at day 7 p.i. (B) Representative dot plots and bar graphs showing the proportions of Th1-like (CXCR3^+^CCR6^-^), Th2-like (CXCR3^-^CCR6^-^) and Th17-like (CXCR3^-^CCR6^+^) Tfh subsets in the spleen of WT and KO mice at day 16 p.i. (C) Representative dot plots and bar graphs showing the proportions and numbers of Tfr (Foxp3^+^CXCR5^+^CD4^+^) cells in the spleen of WT and RACK1 KO mice at day 0 and day 7 p.i. Data are pooled from (A, C) or representative of (B) two or three independent experiments with 6–8 mice/group. Data are shown as mean±SD. ****P<0.0001 and ns, not significant by Student’s *t* test.
(TIF)

S4 Fig CD4^+^ T cell-targeted RACK1 deficiency impairs B cell follicle and GC structures during *P*. *yoelii* infection. (A-B) Representative spleen sections from naïve (day 0) and day16-infected WT and RACK1 KO mice were stained with B220 (green), CD3 (red) and GL7 (pink). Arrows denote typical GC (GL7^+^) structures. Scale bars, 500 μm. (C-E) Summary graphs showing the size of (C) B cell follicles, (D) T cell zones, (E) GC structure areas, and (F) GC numbers per section from the spleen of each mouse. Data are pooled from 3 independent experiments with 5–6 mice/group and are presented as mean±SD. *P<0.05, ****P<0.0001 and ns, not significant by Student’s *t* test.
(TIF)

S5 Fig Identification of STAT3 as RACK1-interacting partner in CD4^+^ T cells after *P*. *yoelii* infection. (A) Splenic CD4^+^ T cell lysates from naïve (D0) WT mice, day 5-infected (D5) WT and KO mice were prepared and IP with anti-RACK1 antibody, the RACK1-immunoprecipitates were subjected to silver staining and mass spectrometric analysis. The arrow denotes STAT3 enrichment in CD4^+^ T cells from day 5-infected WT mice. (B) Peptides of STAT3 identified by mass spectrometry and database searching via the Mascot Daemon 2.4.1 server.
(TIF)

S6 Fig Effects of RACK1 on STAT1, STAT5, ERK1/2, and p38 activation in CD4^+^ T cells after *P*. *yoelii* infection. (A) IB analysis of protein expression and phosphorylation (Tyr^701^) of STAT1 in splenic CD4^+^ T cells from naïve (day 0) and day 5-infected WT and RACK1 KO mice. (B) IB analysis of protein expression and phosphorylation (Tyr^694^) of STAT5 in splenic CD4^+^ T cells at day 0 and day 5 p.i. (C) IB analysis of pERK1/2, p-p38 and ERK1/2, p38 expression in splenic CD4^+^ T cells at day 0 and day 2 p.i. Numbers indicate densitometry of the bands normalized to β-actin, relative to that of uninfected WT mice. Data are representative of two independent experiments with similar results.
(TIF)

S7 Fig Effects of RACK1 on Jak1 expression and serine phosphorylation of STAT3 in CD4^+^ T cells. (A) IB analysis of Jak1 expression in splenic CD4^+^ T cells from WT and RACK1 KO mice at day 5 of *P*. *yoelii* 17XNL infection. Numbers indicate densitometry of the bands normalized to β-actin, relative to that of WT mice. (B) IB analysis of pSTAT3 (Ser^727^) and total amount of STAT3 in splenic CD4^+^ T cells from naïve and day 5-infected WT and RACK1 KO mice. Numbers indicate densitometry of the bands normalized to β-actin, relative to that of uninfected WT mice. Data are representative of two independent experiments with similar results.
(TIF)

S1 Data Excel spreadsheet containing the underlying numerical data for Figs 1D–1F, 2A-2E, 3A–3C, 3E, 4A, 4C–4F, 6B, 6E, 7C–7E, S1, S2, S3, S4 in separate sheets. (XLSX)

S1 Table Antibodies and reagents utilized for flow cytometry and IP/IB. (DOCX)

S2 Table Quantitative RT-PCR primers and shRNA targeting sequences. (DOCX)
