# Supplementary figures and images for: RACK1 enhances STAT3 stability and promotes T follicular helper cell development and function during blood-stage Plasmodium infection in mice
Source: PLoS Pathog. 2024 Jul 18;20(7):e1012352. doi: 10.1371/journal.ppat.1012352 (PMC11288429; doi:10.1371/journal.ppat.1012352)

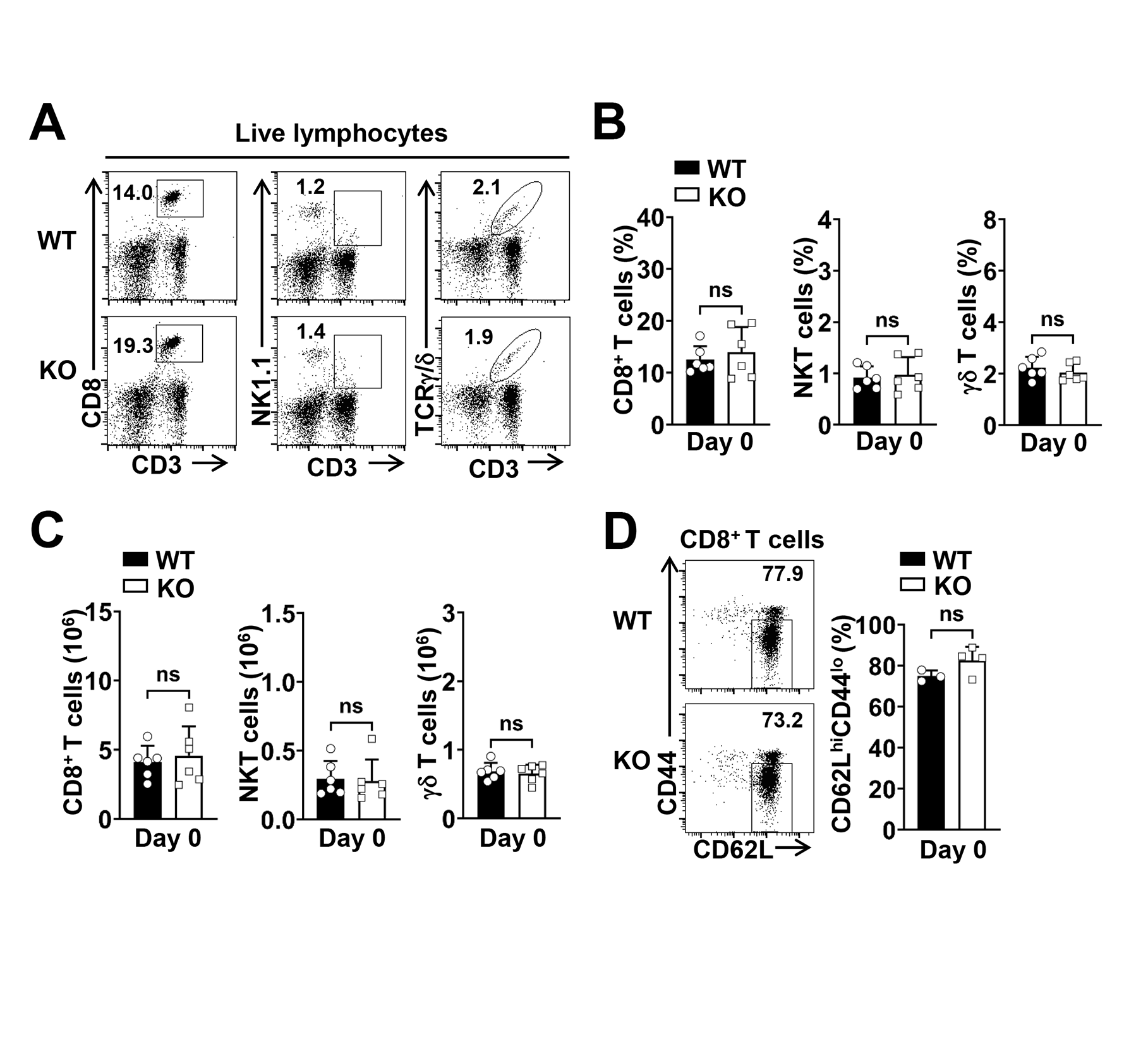

Supplement: S1 Fig — (A) Representative dot plots and (B-C) bar graphs showing the proportions and numbers of CD8+ T (CD3+CD8+), NKT (CD3+NK1.1+), and γδ T (CD3+TCRγ/δ+) cells in the spleen of WT and RACK1 KO mice before P. yoelii 17XNL infection. (D) Representative dot plots and bar graph showing the proportions of naïve (CD62LhiCD44lo) CD8+ T cells in the spleen of WT and RACK1 KO mice before P. yoelii 17XNL infection. Data are pooled from two independent experiments with 3–6 mice/group and are shown as mean±SD. ns, not significant by Student’s t test (B, D) or Mann-Whitney test (C). (TIF) [file ppat.1012352.s001.tif]

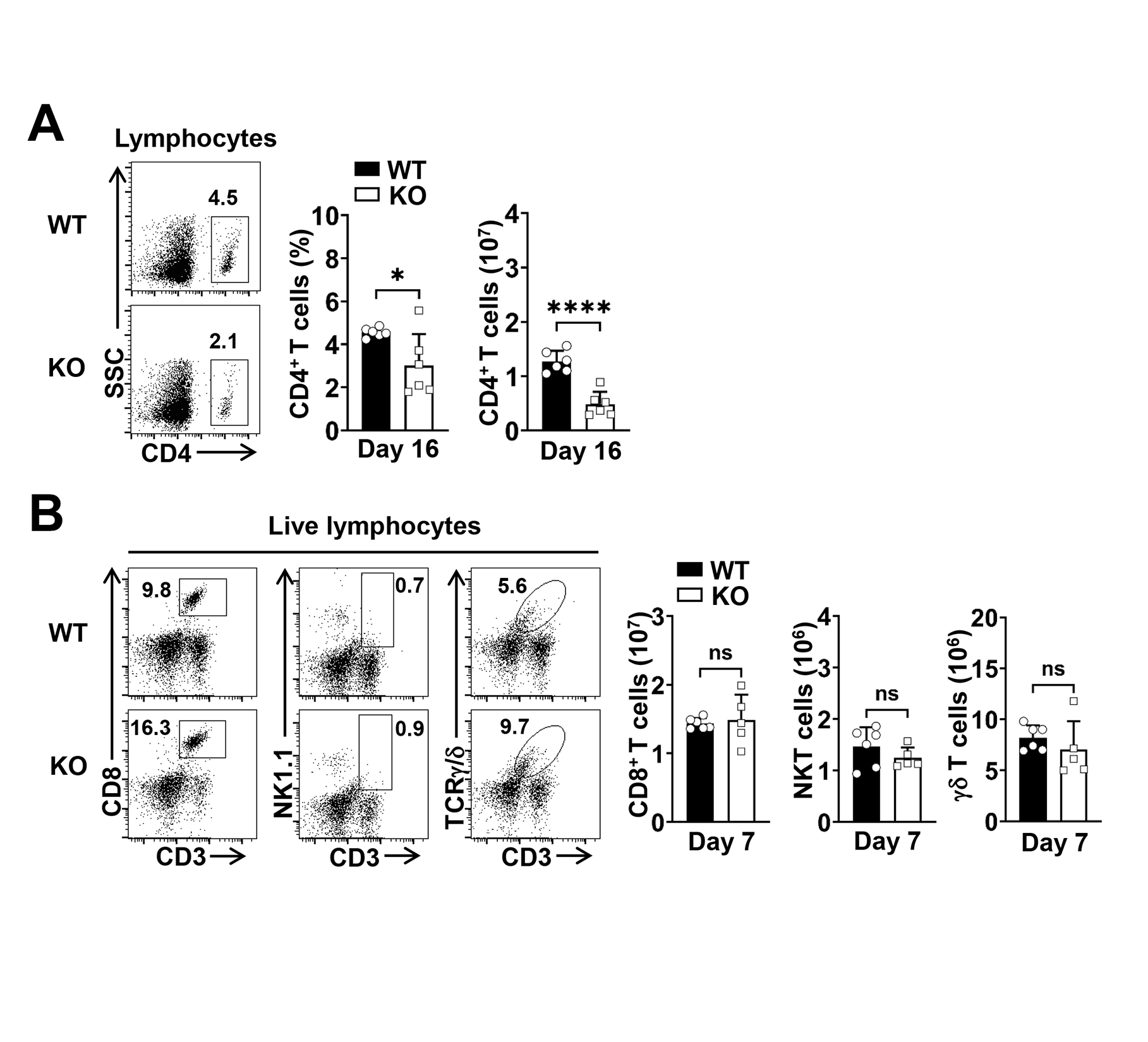

Supplement: S2 Fig — (A) Representative dot plots and bar graphs showing the proportions and numbers of CD4+ T cells in the spleen of WT and RACK1 KO mice at day 16 p.i. (B) Representative dot plots and bar graphs showing the proportions and numbers of CD8+ T cells, NKT cells, and γδ T cells in the spleen of WT and RACK1 KO mice at day 7 p.i. Data are pooled from two independent experiments with 5–6 mice/group and are shown as mean±SD. ns, not significant by Student’s t test. (TIF) [file ppat.1012352.s002.tif]

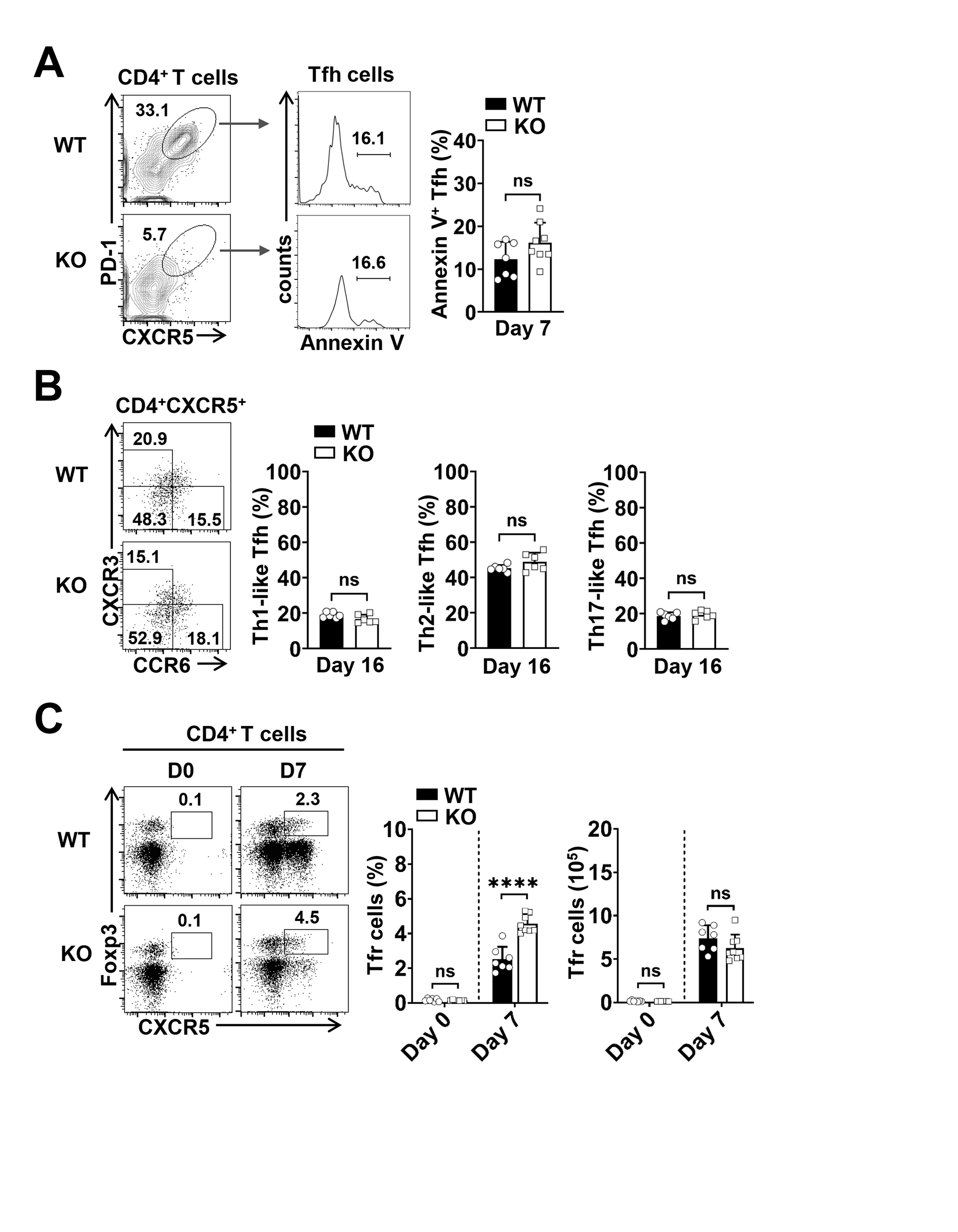

Supplement: S3 Fig — (A) Gating strategy and frequencies of apoptotic (Annexin V+) Tfh cells in the spleen of WT and RACK1 KO mice at day 7 p.i. (B) Representative dot plots and bar graphs showing the proportions of Th1-like (CXCR3+CCR6-), Th2-like (CXCR3-CCR6-) and Th17-like (CXCR3-CCR6+) Tfh subsets in the spleen of WT and KO mice at day 16 p.i. (C) Representative dot plots and bar graphs showing the proportions and numbers of Tfr (Foxp3+CXCR5+CD4+) cells in the spleen of WT and RACK1 KO mice at day 0 and day 7 p.i. Data are pooled from (A, C) or representative of (B) two or three independent experiments with 6–8 mice/group. Data are shown as mean±SD. ****P<0.0001 and ns, not significant by Student’s t test. (TIF) [file ppat.1012352.s003.tif]

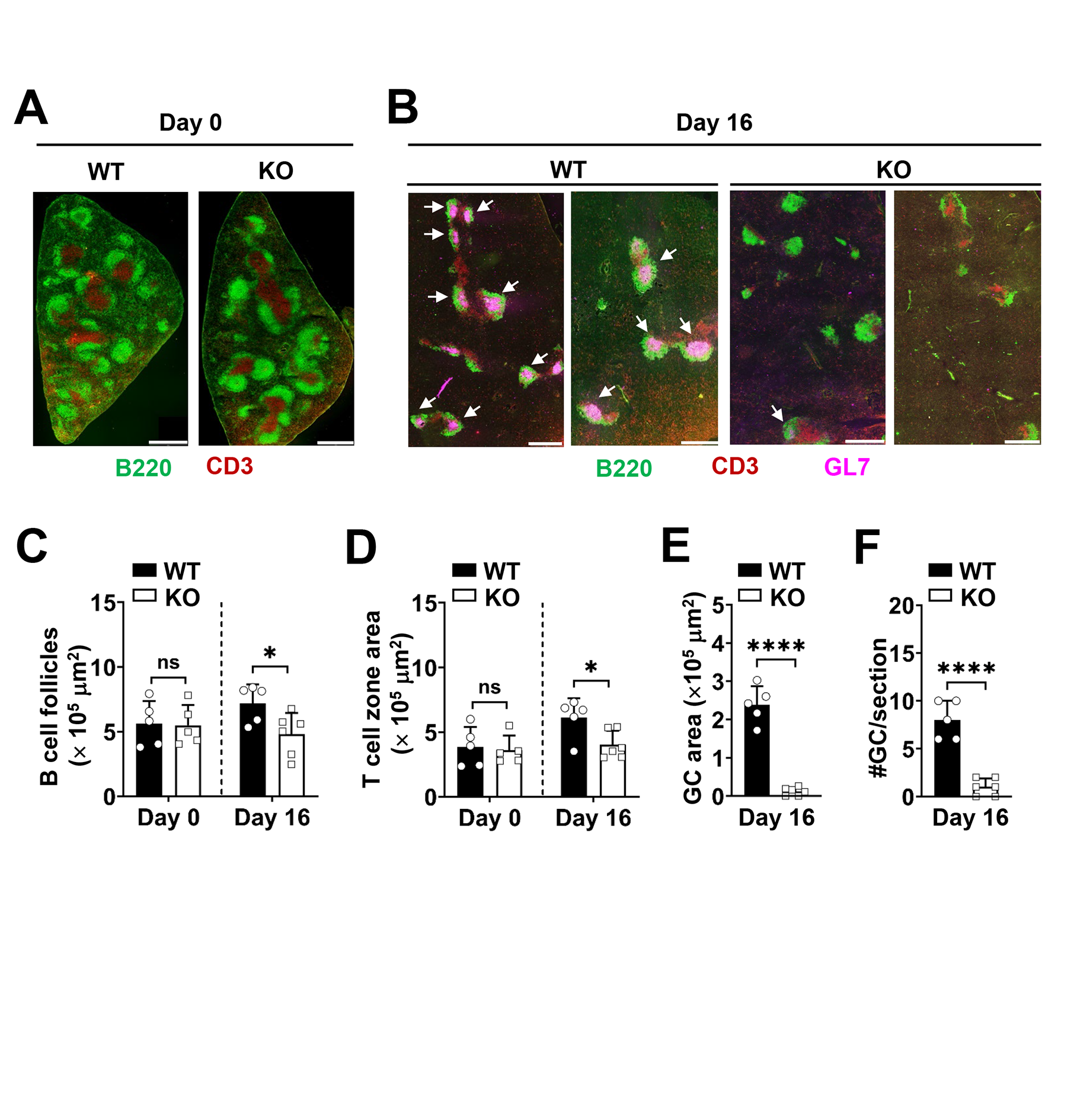

Supplement: S4 Fig — (A-B) Representative spleen sections from naïve (day 0) and day16-infected WT and RACK1 KO mice were stained with B220 (green), CD3 (red) and GL7 (pink). Arrows denote typical GC (GL7+) structures. Scale bars, 500 μm. (C-E) Summary graphs showing the size of (C) B cell follicles, (D) T cell zones, (E) GC structure areas, and (F) GC numbers per section from the spleen of each mouse. Data are pooled from 3 independent experiments with 5–6 mice/group and are presented as mean±SD. *P<0.05, ****P<0.0001 and ns, not significant by Student’s t test. (TIF) [file ppat.1012352.s004.tif]

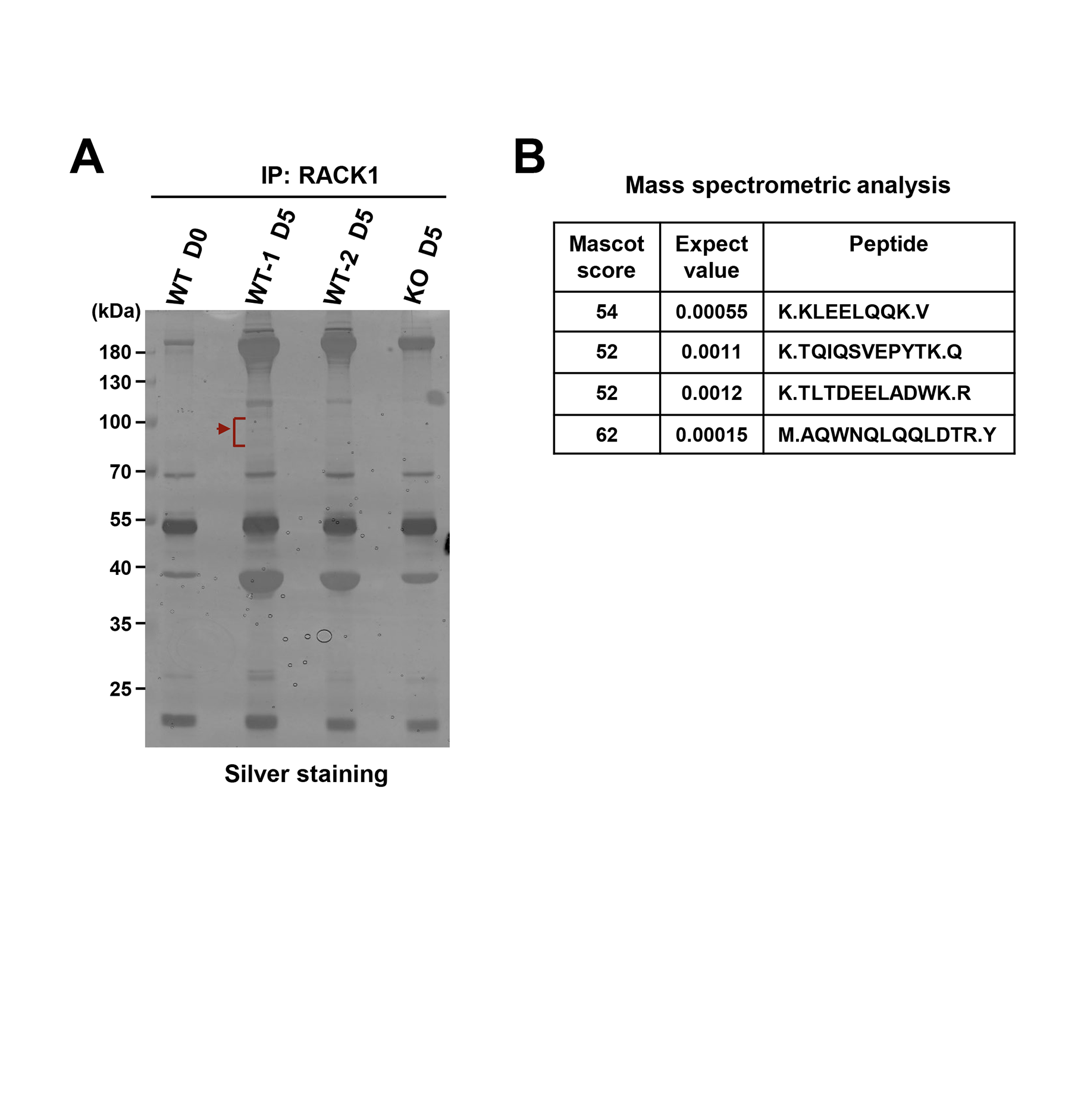

Supplement: S5 Fig — (A) Splenic CD4+ T cell lysates from naïve (D0) WT mice, day 5-infected (D5) WT and KO mice were prepared and IP with anti-RACK1 antibody, the RACK1-immunoprecipitates were subjected to silver staining and mass spectrometric analysis. The arrow denotes STAT3 enrichment in CD4+ T cells from day 5-infected WT mice. (B) Peptides of STAT3 identified by mass spectrometry and database searching via the Mascot Daemon 2.4.1 server. (TIF) [file ppat.1012352.s005.tif]

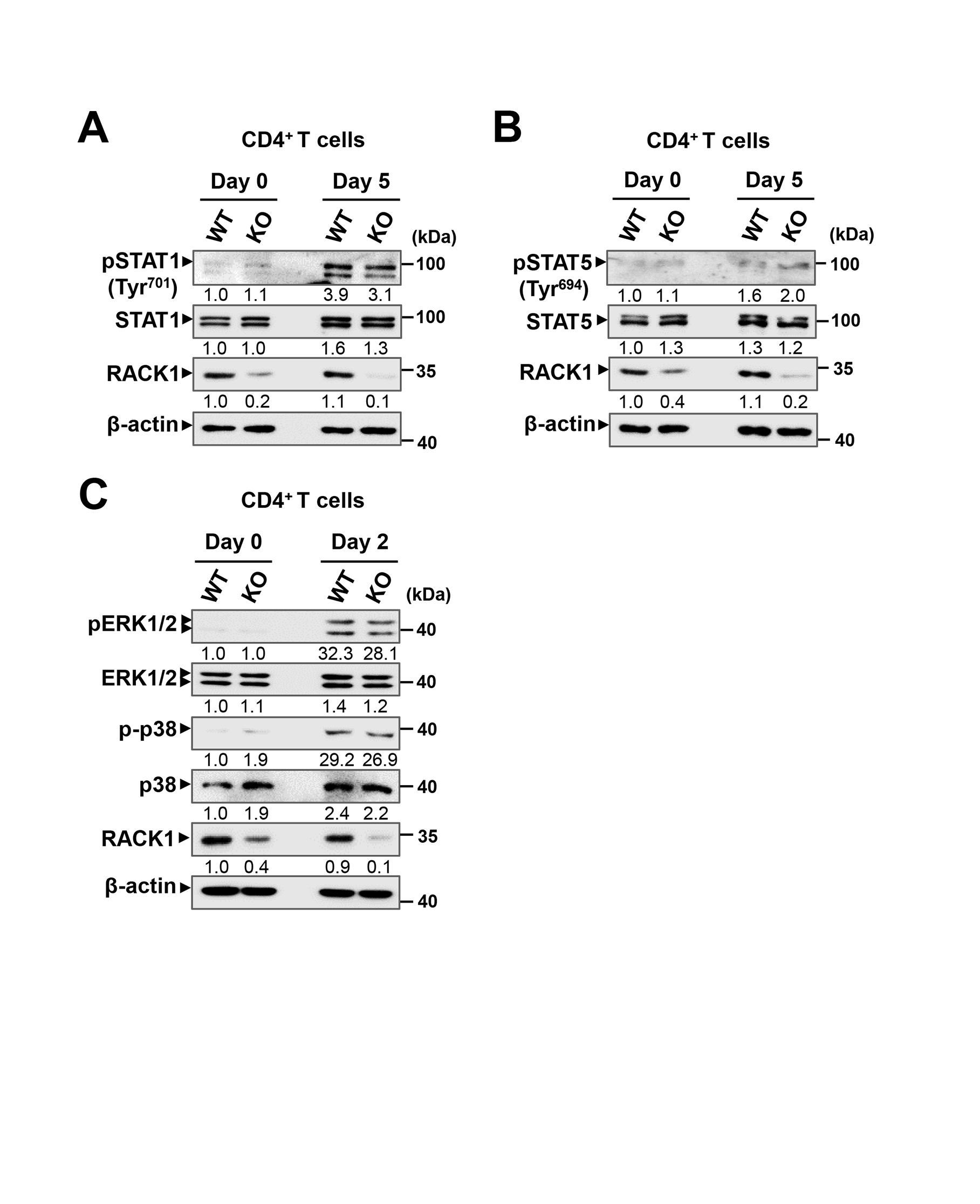

Supplement: S6 Fig — (A) IB analysis of protein expression and phosphorylation (Tyr701) of STAT1 in splenic CD4+ T cells from naïve (day 0) and day 5-infected WT and RACK1 KO mice. (B) IB analysis of protein expression and phosphorylation (Tyr694) of STAT5 in splenic CD4+ T cells at day 0 and day 5 p.i. (C) IB analysis of pERK1/2, p-p38 and ERK1/2, p38 expression in splenic CD4+ T cells at day 0 and day 2 p.i. Numbers indicate densitometry of the bands normalized to β-actin, relative to that of uninfected WT mice. Data are representative of two independent experiments with similar results. (TIF) [file ppat.1012352.s006.tif]

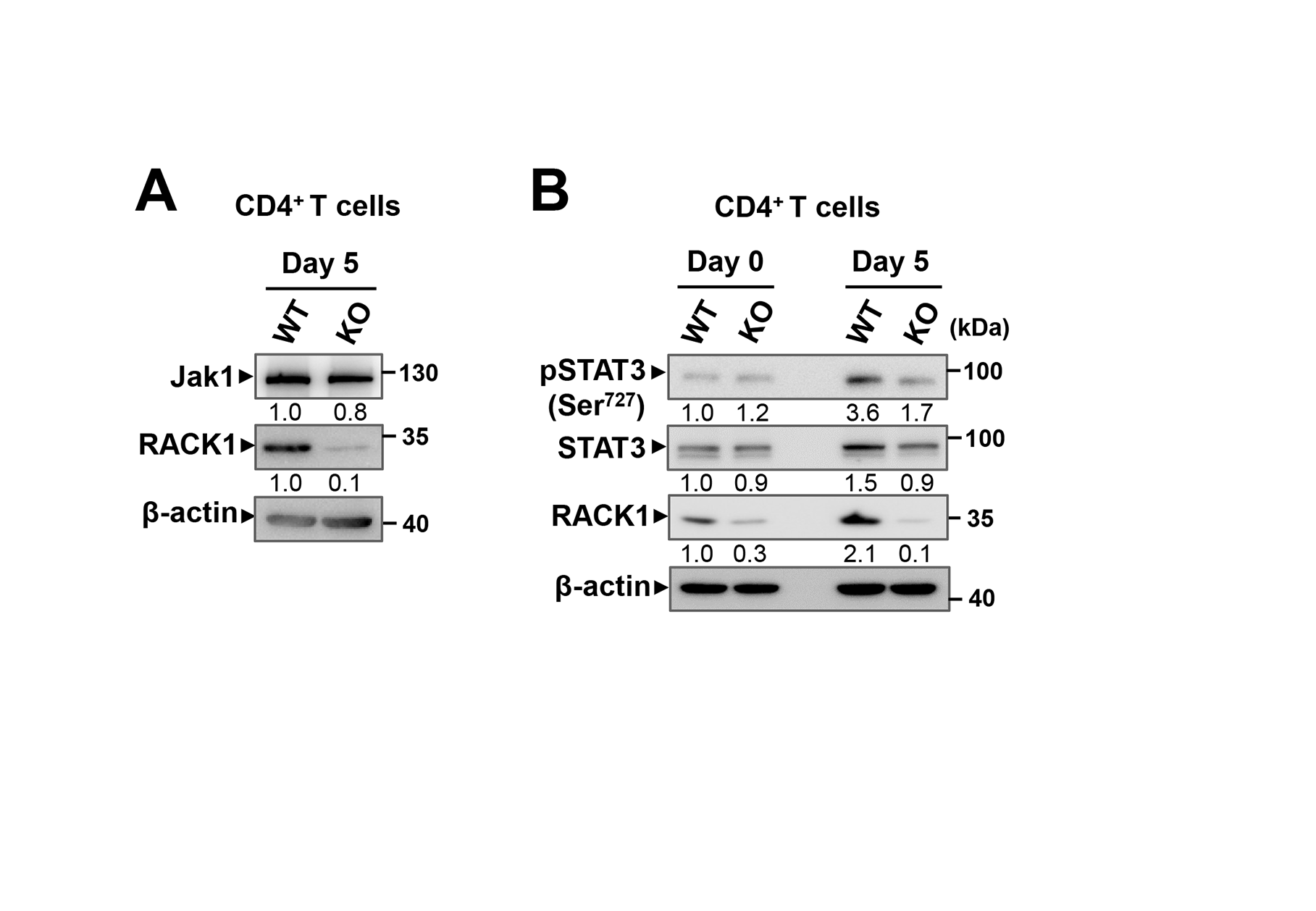

Supplement: S7 Fig — (A) IB analysis of Jak1 expression in splenic CD4+ T cells from WT and RACK1 KO mice at day 5 of P. yoelii 17XNL infection. Numbers indicate densitometry of the bands normalized to β-actin, relative to that of WT mice. (B) IB analysis of pSTAT3 (Ser727) and total amount of STAT3 in splenic CD4+ T cells from naïve and day 5-infected WT and RACK1 KO mice. Numbers indicate densitometry of the bands normalized to β-actin, relative to that of uninfected WT mice. Data are representative of two independent experiments with similar results. (TIF) [file ppat.1012352.s007.tif]
