# Supplementary material for: RACK1 enhances STAT3 stability and promotes T follicular helper cell development and function during blood-stage Plasmodium infection in mice
Source: PLoS Pathog. 2024 Jul 18;20(7):e1012352. doi: 10.1371/journal.ppat.1012352 (PMC11288429; doi:10.1371/journal.ppat.1012352)
Supplement: S1 Table — (DOCX) [file ppat.1012352.s009.docx]

**S1 Table. Antibodies and reagents utilized for flow cytometry and IP/IB.**

| **Antibody/Reagent** | **Source** | **Catalog No.** | **Application** |
| --- | --- | --- | --- |
| Alexa Fluor 488 anti-mouse CD3 (17A2) | Biolegend | 100210 | FC |
| APC anti-mouse CD4 (GK1.5) | Biolegend | 100412 | FC |
| Brilliant Violet 421 anti-mouse CD4 (GK1.5) | Biolegend | 100438 | FC |
| PerCP/Cy5.5 anti-mouse CD8a (53-6.7) | Biolegend | 100734 | FC |
| PE anti-mouse NK1.1 (PK136) | Biolegend | 108708 | FC |
| APC anti-mouse TCRγ/δ (GL3) | Biolegend | 118116 | FC |
| FITC anti-mouse CD4 (GK1.5) | Biolegend | 100406 | FC |
| PE anti-mouse CD62L (MEL-14) | Biolegend | 100408 | FC |
| Brilliant Violet 421 anti-mouse CD44 (IM7) | Biolegend | 103040 | FC |
| PE anti-mouse B220 (RA36B2) | Biolegend | 103208 | FC |
| FITC anti-mouse CD19 (6D5) | Biolegend | 115506 | FC |
| APC anti-mouse CD138 (281-2) | Biolegend | 142506 | FC |
| FITC anti-mouse GL7 (GL7) | Biolegend | 144604 | FC |
| PE anti-mouse CD95 (15A7) | eBioscience | 12-0951-81 | FC |
| FITC Annexin V | eBioscience | 88-8005-72 | FC |
| 7AAD viability staining solution | eBioscience | 00-6993-50 | FC |
| Annexin V Binding buffer | eBioscience | 00-0055-43 | FC |
| Biotinylated anti-mouse CXCR5 (2G8) | BD Pharmingen | 551960 | FC |
| PE streptavidin | BD Pharmingen | 554061 | FC |
| PerCP-eFluor 710 anti-mouse PD-1 (RMP1-30) | eBioscience | 46-9981-82 | FC |
| PerCP/Cy5.5 anti-mouse CXCR3 (CXCR3-173) | Biolegend | 126513 | FC |
| APC anti-mouse CCR6 (29-2L17) | Biolegend | 129813 | FC |
| PerCP/Cy5.5 anti-mouse IgM (RMM-1) | Biolegend | 406512 | FC |
| APC anti-mouse IgD (11-26c.2a) | Biolegend | 405713 | FC |
| PE anti-mouse CD25 (PC61) | Biolegend | 102008 | FC |
| APC anti-mouse Foxp3 (FJK-16s) | eBioscience | 17-5773-82 | FC |
| Brilliant Violet 650 anti-mouse IFN-γ (XMG1.2) | Biolegend | 505832 | FC |
| PerCP/Cy5.5 anti-mouse IL-17A (TC11-18H10.1) | Biolegend | 506920 | FC |
| Brilliant Violet 650 rat IgG1 isotype ctrl (RTK2071) | Biolegend | 400437 | FC |
| PerCP/Cy5.5 rat IgG1 isotype ctrl (RTK2071) | Biolegend | 400425 | FC |
| Alex Fluor 647 mouse anti-pSTAT3 (pY705) | BD Biosciences | 557815 | ICS |
| Alex Fluor 647 mouse IgG2a isotype ctrl | BD Biosciences | 558053 | ICS |
| BD phosflow lyse/fix buffer | BD Biosciences | 558049 | ICS |
| BD Phosflow Perm Buffer II | BD Biosciences | 558052 | ICS |
| STAT3 antibody | Proteintech | 10253-2-AP | ICS |
| Purified mouse anti-Bcl-6 (K112-91) antibody | BD Pharmingen | 561520 | ICS, IB |
| FITC goat anti-mouse IgG secondary antibody | ABclonal | AS001 | ICS |
| FITC goat anti-rabbit IgG secondary antibody | ABclonal | AS011 | ICS |
| phospho-STAT3 (Tyr705) antibody | Cell Signaling Technology | 9131 | IB |
| phospho-STAT3 (Ser727) antibody | Cell Signaling Technology | 9134 | IB |
| STAT3 antibody (D1B2J) | Cell Signaling Technology | 30835 | IP, IB |
| phospho-STAT1 (Tyr701) antibody | Cell Signaling Technology | 9167 | IB |
| STAT1 antibody (1F7C6) | Proteintech | 66545-1-Ig | IB |
| phospho-STAT5 (Tyr694) antibody | Cell Signaling Technology | 9314 | IB |
| STAT5 antibody (D2O6Y) | Cell Signaling Technology | 94205 | IB |
| phospho-ERK1/2 (Thr202/Tyr204) antibody | Cell Signaling Technology | 4370 | IB |
| ERK1/2 antibody (137F5) | Cell Signaling Technology | 4695 | IB |
| phospho-p38 (Thr180/Tyr182) antibody | Cell Signaling Technology | 4511 | IB |
| p38 antibody | Cell Signaling Technology | 9212 | IB |
| Wwp2 antibody (41182) | Proteintech | 12197-1-AP | IB |
| Itch antibody (D8Q6D) | Cell Signaling Technology | 12117 | IB |
| Purified mouse anti-RACK1 antibody | BD Biosciences | 610178 | IB |
| RACK1 antibody (H187) | Santa Cruz Biotechnology | sc-10775 | IP |
| Ubiquitin antibody | Proteintech | 10201-2-AP | IB |
| β-actin antibody (2D4H5) | Proteintech | 66009-1-Ig | IB |
| Myc-tag antibody (My3) | MBL International Corporation | M192-3 | IP/IB |
| Flag-tag antibody (M2) | Sigma-Aldrich | F1804 | IP/IB |
| Goat anti-rabbit IgG-HRP | Santa Cruz biotechnology | sc-2004 | IB |
| Goat anti-mouse IgG-HRP | Santa Cruz biotechnology | sc-2005 | IB |
| IPkine HRP anti-rabbit IgG LCS | Abbkine | A25022 | IB |
| IPkine HRP anti-mouse IgG LCS | Abbkine | A25012 | IB |

FC, flow cytometry; ICS, intracellular staining; IP, immunoprecipitation; IB, immunoblotting.
