# Supplementary material for: RACK1 enhances STAT3 stability and promotes T follicular helper cell development and function during blood-stage Plasmodium infection in mice
Source: PLoS Pathog. 2024 Jul 18;20(7):e1012352. doi: 10.1371/journal.ppat.1012352 (PMC11288429; doi:10.1371/journal.ppat.1012352)
Supplement: S2 Table — (DOCX) [file ppat.1012352.s010.docx]

**S1 Table. Quantitative RT-PCR primers and shRNA target sequences.**

| **Genes** | **Primer sequences** |
| --- | --- |
| *Bcl-6* | Forward: 5’-CTTCCGCTACAAGGGCAAC-3’ |
| *Bcl-6* | Reverse: 5’-GGAAGCGAGTGCCACAGAT-3’ |
| *Cxcr5* | Forward: 5’-GAAAACGAAGCGGAAACTAGAG-3’ |
| *Cxcr5* | Reverse: 5’-ACGCCCACGTAACACCATC-3’ |
| *Pdcd1* | Forward: 5’-TTCTGTGCCTGGAAATGGAG-3’ |
| *Pdcd1* | Reverse: 5’-ACAGCAGATACGCTCACGGA-3’ |
| *Il21* | Forward: 5’-TTCCCTGGAGTGGTATCATCG-3’ |
| *Il21* | Reverse: 5’-GCTATCTTAGGGCGGTACACG-3’ |
| *Icos* | Forward: 5’-AGAATGGTGCCTATTTCCTATGG-3’ |
| *Icos* | Reverse: 5’-CACTCCCGAGACAGGTCAATC-3’ |
| *Tcf7* | Forward: 5’-TACTATGAACTGGCCCGCAA-3’ |
| *Tcf7* | Reverse: 5’-ACTGTCATCGGAAGGAACGG-3’ |
| *Prdm1* | Forward: 5’-TTCGGTCCTGTATTCTCCTAAGC-3’ |
| *Prdm1* | Reverse: 5’-CAAGATGCTACACACCCTCACC-3’ |
| *Sh2d1a* | Forward: 5’-GAAACAGGTTCTTGGAGTGCC-3’ |
| *Sh2d1a* | Reverse: 5’-GTCACGATGCCTTGATCCG-3’ |
| *S1pr1* | Forward: 5’-AGTTGTTGGGAATTTGGTAGGC-3’ |
| *S1pr1* | Reverse: 5’-TTAACTCTCACATTTCACCTCATGC-3’ |
| *Stat3* | Forward: 5’-CCATCCTAAGCACAAAGCCC-3’ |
| *Stat3* | Reverse: 5’-TCTTGCCACTGATGTCCTTTTC-3’ |
| *β-actin* | Forward: 5’-TAAGGCCAACCGTGAAAAGAT-3’ |
| *β-actin* | Reverse: 5’-GGAGAGCATAGCCCTCGTAGAT-3’ |
| *Wwp2* | Forward: 5’- GTGAGAACATCCGAGTTACCG-3’ |
| *Wwp2* | Reverse: 5’- GCCAGTCGCTCATGTCTATCT-3’ |
| *Itch* | Forward: 5’- ACTGATAGCAACGGCAGAGTGTA-3’ |
| *Itch* | Reverse: 5’- GCCCATTGTCTAAGGCTGATTT-3’ |
| sh-*Wwp2* | GCCTTGAGTGTGTCCTCAAAT (target sequence) |
| sh-*Itch* | GCTCCTTGCCACCAACAAATA (target sequence) |
